# Publications as predictors of racial and ethnic differences in NIH research awards

**DOI:** 10.1371/journal.pone.0205929

**Published:** 2018-11-14

**Authors:** Donna K. Ginther, Jodi Basner, Unni Jensen, Joshua Schnell, Raynard Kington, Walter T. Schaffer

**Affiliations:** 1 Department of Economics and Center for Science, Technology & Economic Policy, Institute for Policy & Social Research, University of Kansas, Lawrence, Kansas, United States of America; 2 National Bureau of Economic Research, Cambridge, Massachusetts, United States of America; 3 Discovery Logic/Clarivate Analytics, Rockville, Maryland, United States of America; 4 Grinnell College, Grinnell, Iowa, United States of America; 5 National Institutes of Health, Bethesda, Maryland, United States of America; Indiana University, UNITED STATES

## Abstract

This research expands efforts to understand differences in NIH funding associated with the self-identified race and ethnicity of applicants. We collected data from 2,397 NIH Biographical Sketches submitted between FY 2003 and 2006 as part of new NIH R01 Type 1 applications to obtain detailed information on the applicants’ training and scholarly activities, including publications. Using these data, we examined the association between an NIH R01 applicant’s race or ethnicity and the probability of receiving an R01 award. The applicant’s publication history as reported in the NIH biographical sketch and the associated bibliometrics narrowed the black/white funding gap for new and experienced investigators in explanatory models. We found that black applicants reported fewer papers on their Biosketches, had fewer citations, and those that were reported appeared in journals with lower impact factors. Incorporating these measures in our models explained a substantial portion of the black/white funding gap. Although these predictors influence the funding gap, they do not fully address race/ethnicity differences in receiving a priority score.

## Introduction

The National Institute of Health (NIH) has worked to characterize reasons for the underrepresentation of certain racial and ethnic groups within the population of Principal Investigators with NIH research grants. Earlier studies documented attrition rates for different groups transitioning through important career stages from high school to faculty attainment [1]. Studies also examined race/ethnicity and gender differences in NIH funding [2–4] identifying large differences for PhDs and smaller differences for MDs at medical schools as well as no additional gender gap. The results of these earlier studies sparked an immediate response from the biomedical research community and the NIH [5, 6]. A recent study has shown that the black/white funding gap at the NIH has persisted using data from 2010–2013: 19% of applications from white investigators were funded during this time period compared to only 11.8% of applications from black investigators [7]. Thus, a more-complete understanding of the black/white funding gap is warranted. This study adds to our evolving understanding of the complex problem of differences related to race/ethnicity in NIH funding by extracting additional details about applicants’ prior education, training, and publications from their biographical sketches submitted at the time of application. Previous studies did not include the full range of information available to reviewers from the biographical sketch and could have contributed to unexplained differences in NIH funding. In the new analysis, we examined whether improved information on undergraduate and postdoctoral training, academic rank, scholarly awards, prior grant activity, publications, and associated bibliometrics available at the time of application help to explain the funding gap for applicants of different races and ethnicities.

To receive NIH funding, grant applications are peer-reviewed to evaluate the significance, innovation, and approach of the proposed research along with the investigator(s) and the research environment. The NIH application consists of several parts: the research plan, the biographical sketches of key personnel, and the budget. Assigned reviewers provide a preliminary score for each application. The most meritorious applications (approximately half of the total) are discussed in detail and provided a final priority (more recently, impact) score. NIH Institutes and Centers determine which highly meritorious applications are ultimately funded based on budgets and scientific priorities [8]. In our previous analysis, we hypothesized that scientists of different races and ethnicities with similar academic records would have similar likelihoods of being awarded research grants, but that was not the case. In response, the NIH Director established a high-level Working Group on Diversity in the Biomedical Research Workforce (WGDBRW), and their report [9] pointed out that potentially important explanatory variables were missing from the previous analysis [2]. The report argued that the ability to distinguish between the competing explanations of the black/white NIH funding gap—application merit, investigator characteristics, or bias in the peer review process—was insufficiently explained by variables included in the analysis, prompting a the need for a more detailed evaluation.

## Data and methods

The current study fills in the missing data by using information from biographical sketches (Biosketches) that accompany each NIH grant application. As before, we approached the data with the null hypothesis that information contained in the application determined the probability of an application receiving a priority score and receiving funding. If this is an accurate depiction of the peer review process, then incorporating previously unexplored measures about the investigator from the Biosketch, including his or her academic record, has the potential to explain more of the observed black/white funding gap. In the event that the additional data do not explain the observed gap, and in the absence of other factors, bias in the peer review process remains an open possibility.

We randomly sampled 600 applications for each of the self-identified race/ethnicity categories (Asian, black, Hispanic and non-Hispanic white) from grant proposals submitted between FY 2003 and FY 2006 that had been included in the original sample of 83,188 first-time (Type 1) R01 grant applications from PhD investigators at US institutions [2]. We were limited to that period of time because of the availability of Biosketch information in the NIH IMPAC II grants data system [10, 11]. Our sample included 2,397 applications from 2,025 unique investigators. Three observations were dropped from the original random sample because these applications had incomplete Biosketches. (Fig A in S1 File) indicated no significant differences in R01 Award probabilities by race between the full population previously described and the subsamples used in this study [2].

As part of the application process, an NIH principal investigator included a Biosketch that lists information, in up to four pages, on education and training, positions and honors, selected peer-reviewed publications, and research support. Information from these 2,397 Biosketches was manually entered into a data collection instrument that coded the following information: academic rank of the applicant at the time of application, undergraduate degrees, PhD degrees, postdoctoral positions, publications, scholarly awards and advisory panel experience, and prior grants. Details of data collection and classification are described in the supplemental material, but we provide an overview of items collected here. Altogether, over one million data items were entered or created from the 2,397 Biosketches.

### Education and training

Information on bachelor’s and doctoral degree institution was previously available for US-trained PhDs, but missing for foreign-trained students [2]. Information on postdoctoral institutions was missing for the entire sample in the earlier study. By coding Biosketches we were able to collect undergraduate, PhD, and postdoctoral institutions for all applicants in the subsample and link the data to the Department of Education Integrated Postsecondary Education System Database (IPEDS) [12]; Carnegie Classification of Institutions of Higher Education [13]; and the Oberlin Group of 50 liberal arts colleges [14] in order to examine factors that might be related to the prestige and selectivity of the applicant’s institutional affiliations. We also assigned an NIH funding rank to the undergraduate, PhD, and postdoctoral institutions based in the US using aggregate NIH funding data for the institutions for the same time period.

We identified 1,302 predoctoral and postdoctoral fellowships on applicant Biosketches as well as 170 traineeships and 220 diversity-related fellowships and scholarships. In addition, we incorporated data from IMPAC II on T32 predoctoral traineeships, T32 postdoctoral traineeships, F31 fellowships, and F32 fellowships, including diversity fellowships [15], for the entire sample used previously [2].

We attempted to identify PhD and postdoctoral advisors from multiple sources, but found that few Biosketches identified PhD or postdoctoral advisors. We had limited success in identifying PhD or postdoctoral advisors by using the last author’s name from the applicant’s publications during the time they were doctoral students or postdoctoral researchers. For the subset of applicants for whom we could identify advisors’ names from other sources, only 56% of the last authors listed on their doctoral and postdoctoral publications were the applicant’s advisors. Thus, our analysis could not control for doctoral and postdoctoral advisors.

### Positions and honors

Biosketch data allowed us to identify the academic rank, affiliation, and professional service of the applicant at the time the application was submitted. Applicants were associated with 3,107 advisory panels, 2,005 professional society memberships, and 121 leadership positions.

### Selected peer-reviewed publications

Each peer-reviewed publication listed on the Biosketch was assigned to the application and used to build the bibliographic record for the application. Nearly 54,000 publications were identified on the Biosketches, and the majority of these publications were matched to bibliographic information from the Clarivate Analytics Web of Science and Journal Citation Reports by article title, journal title, volume, and author names. Publications were assigned to undergraduate, graduate, postdoctoral, and independent researcher career stages using the dates of degree receipt and postdoctoral appointments. For each publication, we collected bibliometric data such as the number of times cited, the number of self-citations, the narrow and broad journal subject category, the impact factor of the journal, the number of coauthors, and the author position of the applicant.

Not all scientific fields have the same publication and citation patterns, so we used field-normalized measures based on the journal subject category of the publication. Citations to a given publication over the first two years were compared to citations to papers in journals in a similar Web of Science journal subject category during the same two years. The publication was then assigned a field-normalized quartile rank in the publication distribution. We identified the percentage of the applicant’s publications that were published in each quartile of the field. In addition, we identified the percentage of papers that were uncited and the percentage of papers where the applicant was either first or last author. We also identified the three most frequent last authors and the three most frequent coauthors, regardless of the order of appearance in the list of authors, on the papers published by the applicant and listed on the Biosketch. We then created bibliometric measures similar to those used for the author’s publications based on the publications of the last authors and coauthors. These bibliometric measures serve as proxies for the professional networks of the applicants.

### Research support

An indicator of prior NIH Grants was an important explanatory variable in previous studies [2–4]. We enhanced these data by the counts of prior non-R01 NIH grants by funding mechanism as well as prior NIH R01 Type 2 (competing renewal) awards from the IMPAC II data system [10]. Applicant Biosketches provided additional information on non-NIH federal grants, foundation grants, private sources, and international grants that were not available in the data used in the earlier papers. The Biosketch data was then combined with the variables from the original studies [2, 16]. Table A in S1 File lists the variables used in the analyses described below.

### Modeling grant success outcomes

We analyzed the probability of receiving an R01 award using probit models to evaluate whether different combinations of Biosketch data explained the funding gap. Our analysis was built on a main model that included controls for age, gender, foreign PhD, and race/ethnicity, and progressed through eight models that added sets of explanatory variables for academic rank, grants, scholarly honors, publications and associated bibliometrics, coauthor publications, journal field, and a full model (Table A in S1 File).

In place of reporting probit coefficients, we report the marginal effect of race/ethnicity relative to whites on the award probability, which is the change in the award probability due to race/ethnicity with other variables evaluated at their mean values. These regression estimates are correlations between race/ethnicity and the probability of receiving an R01 award and should not be interpreted as having a causal impact. Our goal is to determine whether additional covariates from the Biosketch explain (reduce the size) of the NIH funding gap. Many variables included in the analysis are endogenous to the process of obtaining additional NIH funding. Thus, we begin our analysis by only including the exogenous variables in the main model. Education variables will also be exogenous to the NIH funding process, whereas training, positions and honors, publications, and prior research support are all endogenous to the NIH funding process. As a result we estimate multiple specifications in order to examine how the race/ethnicity coefficients change as these endogenous variables are added to the model. Since we are examining differences in NIH funding for multiple race/ethnicity categories, all p-values are adjusted for multiple comparisons using the Bonferroni correction.

The Biosketch data features improved measures of publications and associated bibliometrics. Other than our previous works [2–4] that modeled R01 funding as a function of bibliometric measures, we are not aware of other papers that model funding as a function of bibliometrics. Although one study did calculate a variety of bibliometric measures for a small number of NIH awardees [17]. In this study, we observed more publications using Biosketch data (an average of 22.5 publications per investigator) than in the earlier study that used name-matched data (an average of 17.6 publications per investigator) in the earlier study [2]. After a lengthy evaluation of a variety of bibliometric measures detailed in the supplemental material, we determined that the following bibliometric measures provide the optimal fit and explanatory power: the logarithm of the sum of the journal impact factors for all the applicant’s publications, the percentage of publications that were first-authored, the percentage of papers that were last-authored, the percentage of papers in the top quartile of the field (measured by two-year citations to the paper compared with two-year citations to papers published in the journal subject category), the percentage of uncited papers, and the percentage of the top three coauthors’ papers in the top quartile of the field (measured by two-year citations to the paper compared with two-year citations to papers published in the journal subject category).

Use of the journal impact factor to evaluate individual publications is controversial [18, 19]. However, no measure of research impact is perfect, and the journal impact factor compares favorably to other measures [20]. In addition, the sum of the journal impact factors of publications has been used previously to examine academic research productivity [21, 22]. Essentially, the sum of the impact factors weights the publication by the impact factor of the journal. Thus, a publication in a journal like *Science* (impact factor 34.66 in 2015) is given more weight than a publication in *Human Biology* (impact factor .88 in 2015). Journal impact factors have been criticized because citation distributions of papers in journals are highly skewed [19], and we found that citation distributions of papers listed on an applicant’s Biosketch are also highly skewed. Consistent with this observation, we used the natural log of these bibliometric measures to improve the fit of the model.

After establishing the main results that include publications and bibliometrics, we conduct a number of robustness tests to gain a better understanding of the factors contributing to the race/ethnicity gap in NIH funding. Our analysis examines whether race/ethnicity gaps differ between New and Experienced Investigators, those with MD/PhD and PhD degrees, and the funding rank of the institution. NIH has long recognized that new investigators are less-likely to receive NIH research awards than experienced investigators [23]. NIH policy during our study time frame [24] directed NIH Institutes and Centers (ICs) to maintain the number of funded new investigators at the average rate of the previous five years as a way of promoting independent research careers. A recent study showed that mid-career scientists were facing difficulties in receiving additional NIH funding [25]. Given NIH’s focus on differences in funding outcomes for new and experienced investigators [26], we investigated outcomes using these career milestones.

Our previous analysis examined whether the black/white funding gap differed for MDs and MD/PhDs [3]. We found that the black/white funding gap for MDs and MD/PhDs was smaller than for PhD scientists, and could be explained by whether grants included human subjects. Unlike PhD scientists, MDs often have clinical duties in addition to teaching and research that may affect their ability to publish papers.

In addition, our previous analysis also showed that the NIH funding rank of an institution explained a significant share of the race/ethnicity funding gap [2]. The NIH funding rank is a proxy for the research intensity of the institution, where organizations with higher funding conduct more research. We partitioned institutions into the top 100 and the 101+ NIH funded institutions and included our Biosketch measures of publications to determine whether the funding gap differed according to institution type. Next we compared estimates of the race/ethnicity gap when using name-matched and Biosketch measures of publications and bibliometrics.

We then used our model to examine the effect of a counterfactual policy experiment designed to increase publications. We considered a mentoring intervention that provides feedback and professional development for black investigators related to the publication process. This type of intervention increased the number and quality of publications in a randomized controlled trial for female economists [27]. We assumed that this intervention increases both the quantity and quality of publications for black investigators. To understand the impact of this counterfactual policy experiment, we used our models to predict what would happen if black investigators had the average publication and bibliometric profiles as well as average Type 2 awards that matched R01 awardees.

We also used the timing of publications to identify when black and white researchers’ careers diverged. Finally, the black/white funding gap consists of two parts: the probability of receiving a priority score, and then conditional on the score, the probability of receiving funding. We examined whether our measures of publications narrowed the race/ethnicity gap in receiving a priority score.

## Results

### Probability of receiving NIH funding

Table 1 shows the marginal effects for Asian, black, and Hispanic investigators relative to white investigators from the probit models of receiving an R01 grant. Consistent with our previous analysis we find that the Asian and Hispanic funding gaps could be explained by covariates in our model. Accordingly, we focus on the black/white funding gap in our discussion. The main model includes only covariates for race/ethnicity, gender, age, and foreign PhD and indicates that applications from black investigators are -13.4 percentage points (ppt) (p < .001) less likely to receive an R01 award. Sets of covariates from the Biosketches including information on academic rank, prior non-NIH grants, and scholarly activities are added to the main model (in subsequent columns of Table 1), but the inclusion of these variables does not change the significant black/white funding gap. In results not reported, being a full professor increased the probability of NIH funding by 11.9 ppt (p < .001), but the funding gap remained -12.8 ppt (p < .001). Only prior NIH grants had a significant impact on R01 awards—non-NIH grants had no significant effect. Participating on non-NIH review panels (-3.6 ppt (p < .01)) had negative and significant associations with NIH R01 award probabilities.

**Table 1:** Probit Estimates of NIH R01 award.

		Academic		Scholarly	Publications	Add Field	Add	Add	Full
VARIABLES	Main	Rank	Grants	Activities		Normalized	Coauthor	Pub Field	Model
									
Asian	-0.044	-0.038	-0.038	-0.047*	-0.058**	-0.051	-0.050	-0.038	-0.021
	[0.022]	[0.022]	[0.022]	[0.022]	[0.022]	[0.022]	[0.022]	[0.022]	[0.023]
Black	-0.134***	-0.128***	-0.126***	-0.131***	-0.095***	-0.081**	-0.082**	-0.077**	-0.069**
	[0.020]	[0.020]	[0.020]	[0.020]	[0.022]	[0.023]	[0.022]	[0.022]	[0.022]
Hispanic	-0.039	-0.033	-0.030	-0.038	-0.030	-0.029	-0.026	-0.021	-0.004
	[0.022]	[0.023]	[0.023]	[0.022]	[0.023]	[0.022]	[0.022]	[0.023]	[0.023]
Log Sum of Impact Factors					0.067***	0.056***	0.056***	0.083***	0.044***
					[0.010]	[0.010]	[0.010]	[0.013]	[0.011]
Percentage First Authored					0.091*	0.093*	0.091*	0.080	0.093*
Publications					[0.045]	[0.044]	[0.044]	[0.045]	[0.045]
Percentage Last Authored					0.036	0.027	0.027	0.039	-0.035
Publications					[0.043]	[0.042]	[0.042]	[0.043]	[0.045]
Percentage of Uncited						-0.003*	-0.003*	-0.004*	-0.003*
Publications						[0.001]	[0.001]	[0.001]	[0.001]
Percent Publications Top Quartile of Field						0.002***	0.001	0.000	0.001
						[0.001]	[0.001]	[0.001]	[0.001]
Co-Authors have Publications							0.002***	0.002***	0.002***
Top Quartile of Field							[0.001]	[0.001]	[0.001]
NIH Fund Rank 1–30									0.108***
									[0.032]
NIH Fund Rank 31–100									0.043
									[0.030]
NIH Fund Rank 101–200									0.081*
									[0.036]
Total Prior NIH Grants									0.008*
									[0.003]
NIH Review Committee									0.094***
									[0.020]
Grant includes Human									-0.048**
Subjects									[0.019]
Additional Controls:									
Age	X	X	X	X	X	X	X	X	X
Nativity	X	X	X	X	X	X	X	X	X
Gender	X	X	X	X	X	X	X	X	X
Application Year	X	X	X	X	X	X	X	X	X
Academic Rank (1)		X							
Prior Grants (2)			X						
Scholarly Activities (3)				X					
Publication Field Missing					X	X	X	X	X
Publication Field								X	
NIH Institute (4)									X
Observations	2,397	2,396	2,397	2,397	2,397	2,397	2,397	2,397	2,397

Robust standard errors in brackets.

*** p<0.001

** p<0.01

* p<0.05. Source: NIH IMPAC II, National Science Foundation Doctoral Record File, American Association of Medical Colleges faculty roster, select NIH Biosketches, Web of Science®.

1) Includes controls for Assistant, Associate, Full and Research Faculty. Only Full Professor significant .119 (p < .001).

2) Includes controls for prior grants by funder, role on grants as principal investigator, co-investigator, director. Only NIH grants significant .022 (p < .001).

3) Includes controls for scholarly activities. Only Non-NIH Panel member -0.036 (p < .01) significant).

4) Includes eight of the 27 institutes and centers that have a significant impact on probability of R01 Award.

When we added the logarithm of the sum of the journal impact factors and controls for first and last authorship, the funding gap falls from -13.3 ppt (p < .001) to -9.5 ppt (p < .001). A one percent increase in the sum of the journal impact factors increases the probability of NIH funding by 6.7 ppt (p < .001). Likewise a one percent increase in the percentage of first authored publications increases the probability by 9.1 ppt (p < .05) (Table 1). Field normalized publications, measured by the percentage of papers in the top quartile of the field, and the percentage of uncited papers reduced the gap to -8.1 ppt (p < .01) (Table 1). Although coauthors who publish in the top quartile of the field are positively associated with NIH research awards, this variable does not change the black/white funding gap. Controlling for field of publication reduces the gap to -7.7 ppt. (p < .001). Next, we added important explanatory variables from our previous analysis such as NIH funding rank of the employer and the number of prior NIH grants, and the black/white funding gap fell to -6.9 ppt (p < .01).

We separately analyzed the effect of training variables on the probability of receiving an NIH R01 award. Building on the main model, we progressed through nine models that added sets of explanatory variables for each stage of training: undergraduate, predoctoral, receipt of PhD, postdoctoral, and pre and postdoctoral fellowships (specifications Table B, estimates Table C in S1 File).

Adding undergraduate training, predoctoral fellowships, and variables describing the PhD-granting institution to the main model does not diminish the funding gap for black investigators. Adding postdoctoral characteristics in the last four models reduces the black/white funding gap to about -12.7 ppt (p < .001), and when all training variables are entered into the model, the black/white funding gap is only slightly smaller at -12.3 ppt (p < .001). The final specification limits the analysis to US citizens in order to evaluate the impact of NIH training, and the black/white gap falls to -11.9 ppt (p < .001). These results indicated that bibliometric measures better explain the black/white funding gap when compared to training measures.

Given the importance of publication and bibliometric measures for explaining the funding gap, we used a method developed by Gelbach [28] to estimate the contribution of covariates in our model to the reduction in the black/white funding gap. To implement this method we estimated a linear probability model using the main specification and the Biosketch model that includes all the covariates from the main, rank, grants, scholarly activities, field-normalized publications, and coauthor publications models (see Table A in S1 File for the specification). We omitted the training covariates because they do not appreciably change the race/ethnicity coefficients. Results are reported in the top panel of Table D in S1 File. Linear probability estimates in the main model indicate that black investigators are 14.1 ppt (p < .001) less likely to receive funding than white investigators. Once the covariates from the Biosketch model are included, the gap falls by 40% to 8.4 ppt (p < .001). Almost all of the reduction in the gap is due to the inclusion of bibliometric measures derived from the publications reported on the application at the time of review.

Next we compare the main model to the full model in the bottom panel of Table D in S1 File. The full model uses Biosketch publications and improved information on previous NIH funding (see Table A in S1 File for a complete list of covariates). The full model explains 52% of the black/white funding gap. Half of the explained gap is due to Biosketch publications (p < .001) and an additional 38% is due to improved measures of prior NIH funding and Type 2 awards.

### New and experienced investigators, MD/PhDs and funding rank of the institution

**Fig 1:**
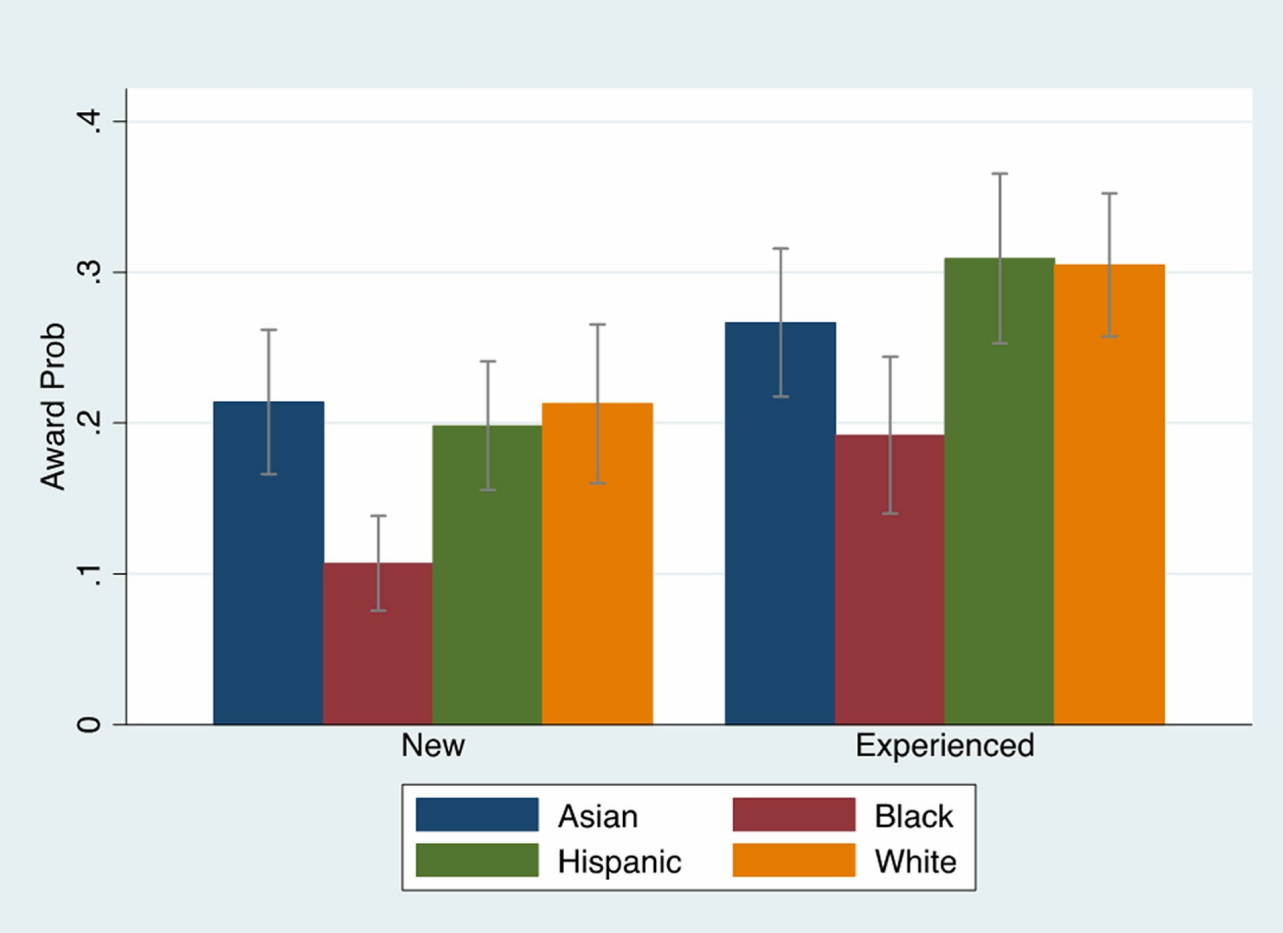
R01 Awards by race and investigator experience. A comparison of R01 award probability by race/ethnicity, by investigator experience with 95% confidence intervals. Source: NIH IMPAC II, National Science Foundation Doctoral Record File, American Association of Medical Colleges faculty roster, select NIH Biosketches, Web of Science. Full Sample N = 53,454. Subsample N = 2,397.

Fig 1 shows the R01 award probability, and Table E in S1 File shows the counts of applications and awards by race for new and experienced investigators. We found that 31% of experienced white investigators were funded compared to only 21% of new white investigators. With the exception of Asians, experienced investigators have an approximately 10 percentage point advantage in NIH research funding. However, the funding disadvantage for black investigators remains significant. Table 2 examined whether the black/white funding gap differed for new and experienced investigators. Using the main model that only controls for demographic characteristics, we found that the black/white funding gap for new investigators was smaller (-10.2 ppt (p < .001)) than for experienced investigators (-12 ppt, (p < .001)). Experienced investigators can receive renewals (Type 2 R01 awards) on their previously funded Type 1 R01 awards. We incorporated this variable in the full model and found that each additional Type 2 award increased the probability of receiving a new Type 1 R01 award by 2 ppt (p < .01), but it did not change the overall black/white funding gap. We tested whether the factors that predicted NIH research awards differ significantly between new and experienced investigators in our probit models, and rejected the null hypothesis that the coefficients were the same (p < .04). The black/white funding gap falls to 4.6 ppt. (p = .33) for new investigators. However, the black/white funding gap for experienced investigators is almost double that at 7.8 ppt. (p = .13) (Table 2, Fig 1).

**Table 2:** Probit Estimates of NIH R01 Award by investigator experience.

	Main	Main	Main	Full	Full +	New	Experienced
VARIABLES		New Investigator	Experienced Investigator		Type 2	Investigator	Investigator
							
Asian	-0.044*	-0.010	-0.063	-0.021	-0.018	-0.001	-0.027
	[0.022]	[0.031]	[0.033]	[0.023]	[0.023]	[0.029]	[0.035]
Black	-0.134***	-0.102***	-0.120**	-0.069**	-0.068*	-0.046	-0.078
	[0.020]	[0.026]	[0.034]	[0.022]	[0.022]	[0.027]	[0.036]
Hispanic	-0.039	-0.024	-0.023	-0.004	-0.004	-0.005	0.001
	[0.022]	[0.030]	[0.035]	[0.023]	[0.023]	[0.028]	[0.036]
Log Sum of Impact Factors				0.044***	0.043***	0.043***	0.042*
				[0.011]	[0.011]	[0.012]	[0.020]
Percentage First Authored				0.093*	0.098*	0.049	0.140
Publications				[0.045]	[0.044]	[0.049]	[0.079]
Percentage Last Authored				-0.035	-0.044	-0.005	-0.034
Publications				[0.045]	[0.045]	[0.062]	[0.066]
Percentage of Uncited				-0.003*	-0.003*	-0.004**	-0.001
Publications				[0.001]	[0.001]	[0.001]	[0.003]
Percent Publications Top Quartile of Field				0.001	0.001	0.000	0.001
				[0.001]	[0.001]	[0.001]	[0.001]
Co-Authors have Publications				0.002***	0.002***	0.002**	0.002**
Top Quartile of Field				[0.001]	[0.001]	[0.001]	[0.001]
NIH Fund Rank 1–30				0.108***	0.107***	0.128**	0.052
				[0.032]	[0.032]	[0.041]	[0.051]
NIH Fund Rank 31–100				0.043	0.041	0.067	-0.016
				[0.030]	[0.030]	[0.036]	[0.049]
NIH Fund Rank 101–200				0.081*	0.078*	0.077	0.049
				[0.036]	[0.036]	[0.042]	[0.059]
Total Prior NIH Grants				0.008*	-0.001	0.004	-0.006
				[0.003]	[0.005]	[0.011]	[0.006]
NIH Review Committee				0.094***	0.099***	0.073**	0.105***
				[0.020]	[0.020]	[0.028]	[0.030]
Grant includes Human				-0.048**	-0.042*	-0.026	-0.057
Subjects				[0.019]	[0.019]	[0.023]	[0.031]
Type 2 Award					0.020**		0.027**
					[0.007]		[0.009]
Observations	2,397	1,232	1,165	2,397	2,397	1,232	1,165

Robust standard errors in brackets.

*** p<0.001

** p<0.01

* p<0.05.

Source: NIH IMPAC II, National Science Foundation Doctoral Record File, American Association of Medical Colleges faculty roster, select NIH Biosketches, Web of Science.

The new and experienced investigator estimates must be interpreted with care. It is important to note that our sample contains almost all of the black investigators who submitted applications between 2003 and 2006, and this is the maximum possible sample size. When we split the sample into new and experienced investigators, the standard errors became larger, making it more likely to commit a Type 2 error (failing to reject the null hypothesis of no black/white funding difference when it is false and there is a difference). There are 150 more black new investigators than experienced investigators, but the p-value for new investigators is much larger than for experienced investigators, suggesting that the remaining variation is sample noise. Since there are a smaller number of black experienced investigators, we would expect to have less precise estimates resulting in larger standard errors and p-values for experienced compared to new investigators. The smaller p-value for experienced investigators contradicts this intuition.

We observed considerable evidence that the factors associated with new investigator funding differ from those associated with experienced investigator funding (Table 2). In particular, the percentage of uncited papers had a larger negative effect on new investigators. Likewise, the NIH funding rank of the employer and prior NIH grants, increased the probability of research funding for new, but not experienced investigators. The coefficient on NIH review committee experience is positive and significant for both new (7.3 ppt, p < .01) and experienced (10.5 ppt, p < .001) investigators but it is much larger for the experienced PIs. Investigators eligible to obtain renewals on their previous R01 awards (R01 Type 2 awards) are by definition, experienced investigators. Each additional Type 2 award increases the probability that experienced investigators receive funding for a new R01 project by 2.7 ppt (p < .001).

We partitioned our data into MD/PhDs and MDs as well as Top 100, and 101+ NIH funded institutions to see whether Biosketch publications narrowed the race/ethnicity funding gap in these subsamples. Table 3 shows the race/ethnicity coefficients for the main model that only includes exogenous variables and the full model. In the main model, the estimate of the black/white funding gap is remarkably similar in magnitude and statistically significant for the full sample, MD/PhDs, PhDs, 101+ NIH Funded, and Top 100 institutions. The sample size of MD/PhDs is small (N = 476) as is the sample of 101+ NIH Funded institutions (N = 763). When we included the covariates in the full model, the black/white funding gap for MD/PhDs drops by half compared to the full sample (-3.8 ppt compared with -6.8 ppt) and is no longer statistically significant. However, the black/white funding gap for PhDs is larger than the coefficient for the full sample (-7.6 ppt, p < .01). The black/white funding gap for the NIH Funding rank subsamples are not statistically significant, but the magnitude suggests economic significance. The black/white funding gap for the 101+ NIH funded institutions is -7.4 ppt while the gap is -5.5 ppt for the top 100 NIH funded institutions.

**Table 3:** Probit Estimates of NIH R01 Award by degree and NIH funding rank.

	Main Model					Full Model				
VARIABLES	Full Sample	MD/PhDs	PhDs	101+ NIH Funding	Top 100 NIH Funding	Full Sample	MD/PhDs	PhDs	101+ NIH Funding	Top 100 NIH Funding
Asian	-0.044	-0.140*	-0.024	-0.020	-0.051	-0.018	-0.116*	0.005	-0.012	-0.018
	[0.022]	[0.048]	[0.025]	[0.037]	[0.028]	[0.023]	[0.048]	[0.026]	[0.036]	[0.029]
Black	-0.134***	-0.132*	-0.139***	-0.127***	-0.124***	-0.068*	-0.038	-0.076**	-0.074	-0.055
	[0.020]	[0.047]	[0.021]	[0.031]	[0.026]	[0.022]	[0.055]	[0.024]	[0.033]	[0.030]
Hispanic	-0.039	-0.049	-0.043	0.020	-0.063	-0.004	-0.026	0.000	0.035	-0.022
	[0.022]	[0.053]	[0.025]	[0.040]	[0.027]	[0.023]	[0.051]	[0.026]	[0.040]	[0.029]
Observations	2,397	476	1,921	763	1,634	2,397	476	1,921	763	1,634

Robust standard errors in brackets.

*** p<0.001

** p<0.01

* p<0.05.

Source: NIH IMPAC II, National Science Foundation Doctoral Record File, American Association of Medical Colleges faculty roster, select NIH Biosketches, Web of Science

### A comparison of name-matched and Biosketch publications

In our previous studies we included measures of publications and citations, but these variables did not fully explain the black/white funding gap. As discussed above, publications were identified in the previous study using a name-matching algorithm, a standard approach for assigning publications to applicants. However, this resulted in an undercount of publications. Estimates in Table 4 demonstrated that 2 percentage points of the -13.4 ppt (p < .001) black/white funding gap could be explained without including measures of publications. Our name-matched publication measures from the previous study did not include the log sum of journal impact factors. Instead, we included the log of publications, the log of citations, the maximum journal impact factor on all publications, the median journal impact factor on all publications, and the percentage of first, last, and single-authored publications from the name-matched measures. The name-matched publication variables explain 0.8 ppt of the black/white funding gap. We can replicate these measures with our Biosketch publication variables, and the black/white funding gap reduces from -13.4 to -8.8 ppt (p < .05). We replaced these measures with our new measures that included the log sum of the impact factors of publications, and the black/white funding gap drops from -13.4 to -8.6 ppt (p < .001).

**Table 4:** Probit Estimates of NIH R01 Award comparing name-matched and Biosketch publications.

	Main	Main +	Name-matched	Biosketch	Biosketch Pubs &
VARIABLES		Covariates	Publications	Publications	New Measures
					
Asian	-0.044*	-0.019	-0.027	-0.016	-0.028
	[0.022]	[0.023]	[0.023]	[0.041]	[0.023]
Black	-0.134***	-0.110***	-0.103***	-0.088*	-0.086***
	[0.020]	[0.020]	[0.021]	[0.043]	[0.021]
Hispanic	-0.039	-0.012	-0.010	-0.024	-0.008
	[0.022]	[0.023]	[0.023]	[0.040]	[0.023]
NIH Fund Rank 1–30		0.143***	0.128***	0.142**	0.119***
		[0.032]	[0.032]	[0.051]	[0.032]
NIH Fund Rank 31–100		0.071*	0.060*	0.094	0.053
		[0.030]	[0.030]	[0.051]	[0.030]
NIH Fund Rank 101–200		0.093*	0.087*	0.123*	0.082*
		[0.037]	[0.037]	[0.058]	[0.036]
Prior NIH Grants		0.064**	0.052*	0.059	0.051*
		[0.021]	[0.022]	[0.042]	[0.021]
NIH Review Committee		0.102***	0.098***	0.082*	0.095***
		[0.019]	[0.020]	[0.035]	[0.020]
Grant includes Human		-0.069***	-0.056**	-0.103**	-0.050**
Subjects		[0.018]	[0.019]	[0.034]	[0.019]
Log Publications			-0.003	0.002	
			[0.013]	[0.024]	
Log Citations			0.006	0.029*	
			[0.009]	[0.013]	
Maximum Impact Factor			0.002	-0.000	
			[0.001]	[0.001]	
Median Impact Factor			0.007*	0.012**	
			[0.003]	[0.004]	
Percentage First Authored			-0.041	0.086	0.098*
Publications			[0.035]	[0.045]	[0.045]
Percentage Last Authored			-0.030	-0.009	-0.030
Publications			[0.037]	[0.044]	[0.044]
Percentage of Single-Authored			-0.046	-0.077	
Publications			[0.052]	[0.093]	
Log Sum of Impact Factors					0.042***
					[0.011]
Percentage of Uncited					-0.003*
Publications					[0.001]
Percent Publications Top Quartile of Field					0.001
					[0.001]
Co-Authors have Publications					0.002***
Top Quartile of Field					[0.001]
Additional Controls:					
Age	X	X	X	X	X
Nativity	X	X	X	X	X
Gender	X	X	X	X	X
Application Year	X	X	X	X	X
Publication Field Missing				X	X
NIH Institute (1)		X	X	X	X
Observations	2,397	2,397	2,397	2,397	2,397

Robust standard errors in brackets.

*** p<0.001

** p<0.01

* p<0.05.

Source: NIH IMPAC II, National Science Foundation Doctoral Record File, American Association of Medical Colleges faculty roster, select NIH Biosketches, Web of Science.

1) Includes eight of the 27 institutes and centers that have a significant impact on probability of R01 Award.

Why do bibliometric measures based on the journal impact factor have better explanatory power than citation counts of an individual’s publication record? Journals are observed on the Biosketch, and citations are not. Furthermore, review panel members are likely to be familiar with the impact factors of journals in their fields. Reviewers appear to be using their understanding of the journal impact factor as a proxy for the quality of the publication, despite warnings to avoid this attribution [18].

### Race/Ethnicity differences in publications and bibliometrics

**Fig 2:**
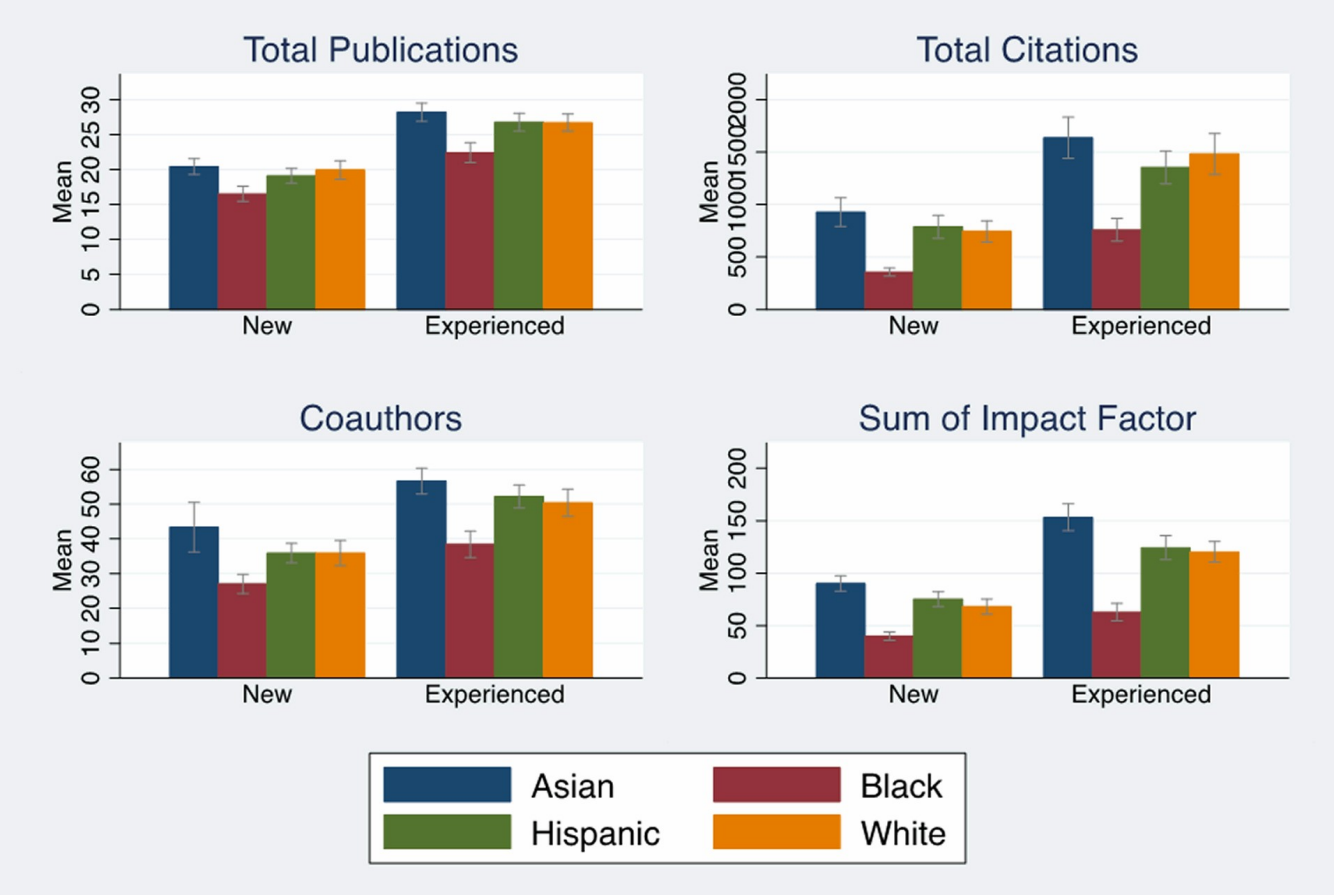
Average productivity by race, experience. Average publications, citations, coauthors, and sum of impact factors by race and investigator experience with 95% confidence intervals. Source: NIH IMPAC II, National Science Foundation Doctoral Record File, American Association of Medical Colleges faculty roster, select NIH Biosketches, Web of Science. N = 2,397.

**Fig 3:**
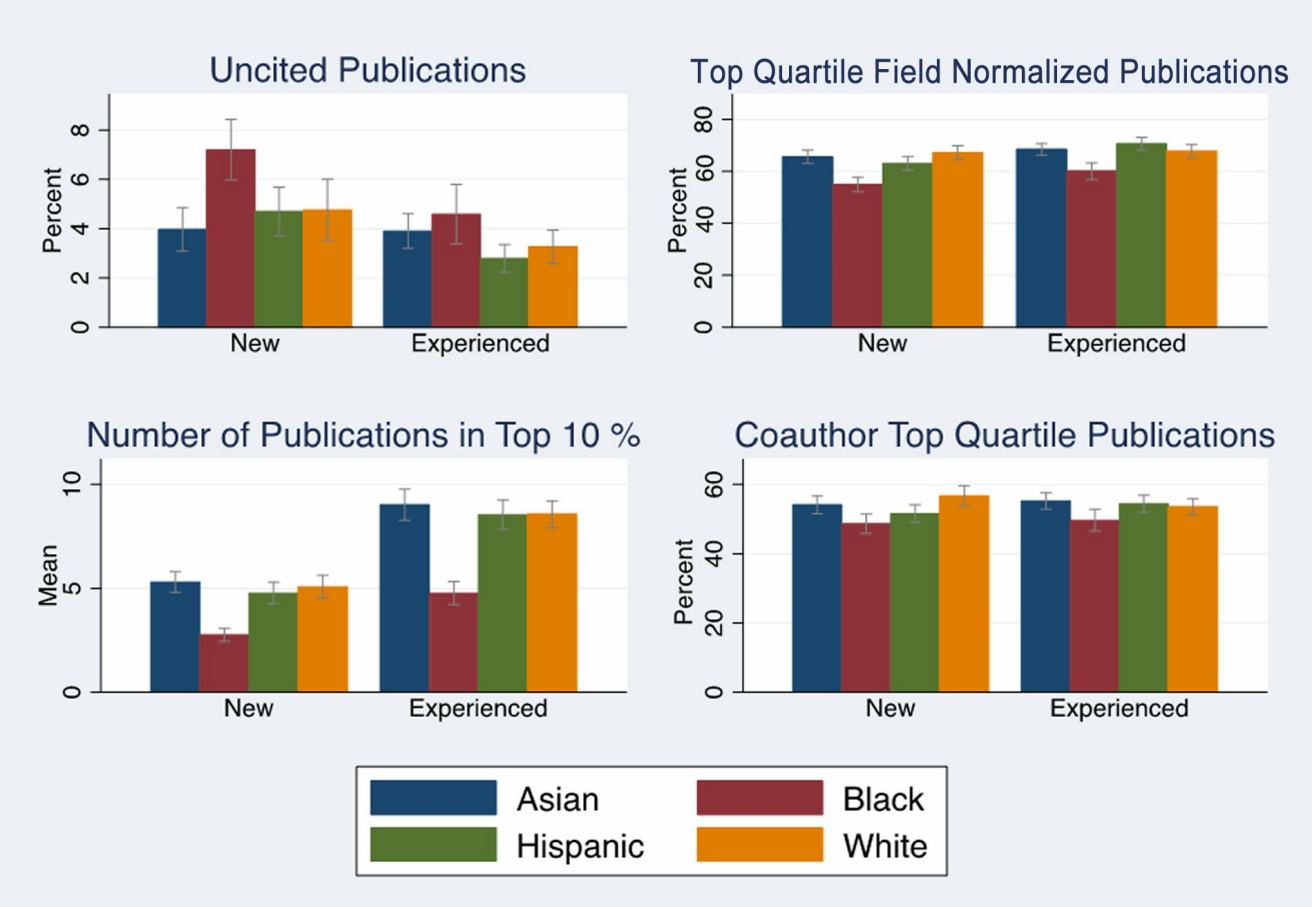
Relative productivity by race, experience. Measures of uncited publications, field normalized publications, and coauthor publications of NIH R01 applications by race/ethnicity, 2003–2006 with 95% confidence intervals. Source: NIH IMPAC II, National Science Foundation Doctoral Record File, American Association of Medical Colleges faculty roster, select NIH Biosketches, Web of Science. N = 2,397.

Unlike our previous studies, publications and the associated bibliometrics explained a significant portion of the black/white funding gap, suggesting the need to explore factors that may help us understand the differences in publication-related metrics. Figs 2 and 3 (Tables F and G in S1 File) examined differences in average publications and associated bibliometrics by race, R01 award status and investigator experience. Fig 2 (Tables F and G in S1 File) showed that new and experienced black investigators report between two to three fewer papers than whites, and those papers are cited at less than half the frequency of whites for non-awardees. Black investigators also have fewer coauthors (Tables F and G in S1 File), and their reported publications have lower average sums of impact factors. These gaps are larger for non-awarded applicants than for those who receive an R01 award, and black non-awardees have significantly lower sum of impact factors than white non-awardees (p < .001). Fig 3 indicates that a higher percentage of reported papers from black new and experienced applicants were uncited; a lower percentage of reported papers from new and experienced black applicants were published in the top quartile and the top 10% of the field (measured by citations relative to citations to papers in the journal subject category). Fig B in S1 File shows that black experienced investigators have had one Type 2 award compared to two Type 2 awards for white experienced investigators.

### Counterfactual impact of publications on R01 awards

**Fig 4:**
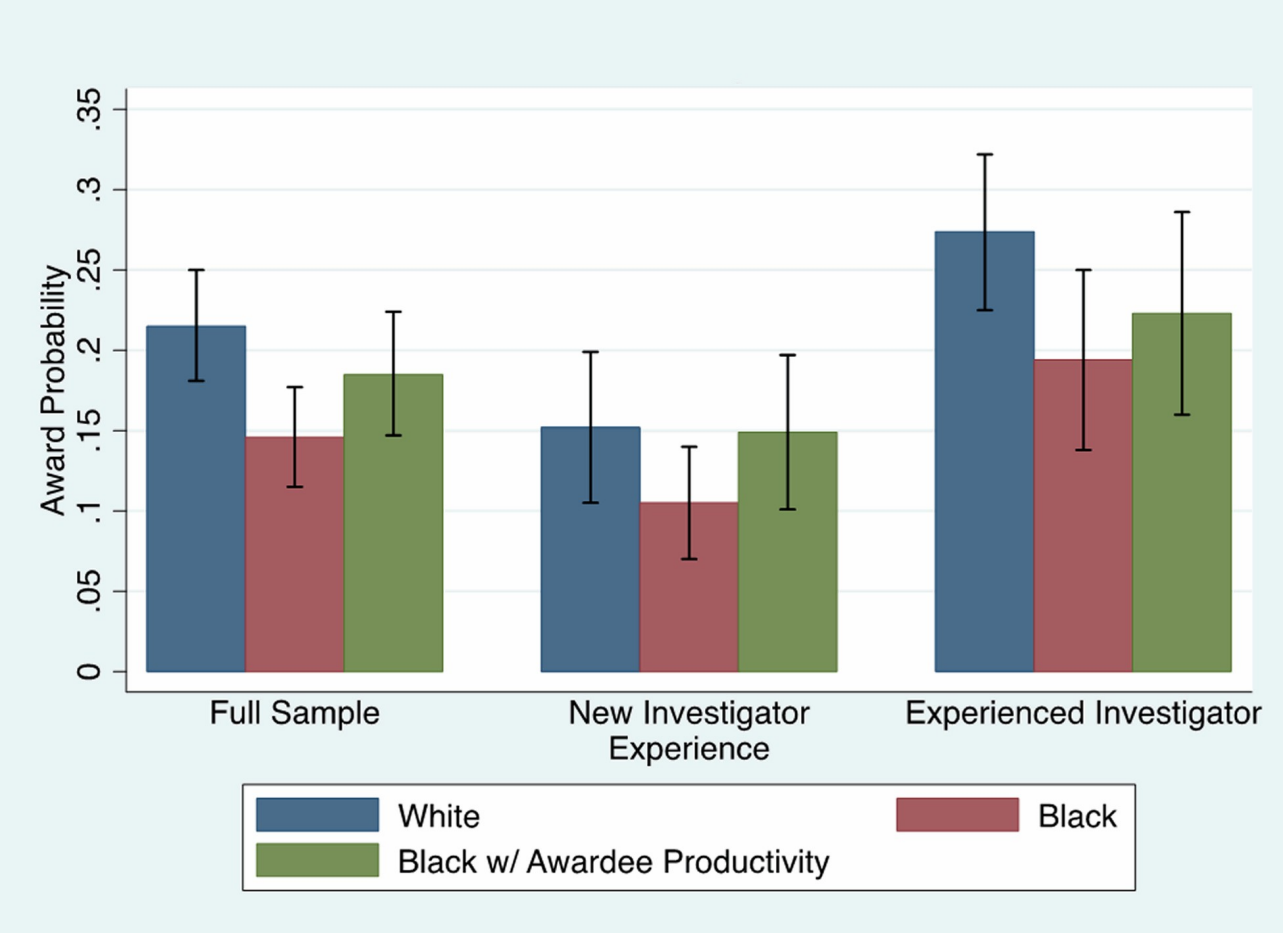
Predicted probability of R01 award. Predicted probability of NIH R01 awards by race and investigator experience with 95% confidence intervals. Source: NIH IMPAC II, National Science Foundation Doctoral Record File, American Association of Medical Colleges faculty roster, select NIH Biosketches, Web of Science. N = 2,397.

Our analysis has shown that publications explain about half of the black/white funding gap. Figs 2 and 3 (Fig C, Tables F and G in S1 File) show there are large and statistically significant differences in publications and associated bibliometrics. We now use our model to examine the effect of a counterfactual policy experiment designed to simulate an increase in publication quantity and quality. We used our models to predict what would happen if black investigators had the publication and bibliometric profiles as well as average Type 2 awards that matched R01 awardees. Fig 4 shows the predicted probability of receiving an NIH R01 award conditional on having the average characteristics of the sample for all other variables except for being white (blue bar) and black (red bar). The difference between these two bars is the estimated effect of being black on the probability of receiving NIH funding in Table 1. The green bar is a counterfactual analysis for black investigators where the model is estimated at the means of the sample for all variables except publications, bibliometrics, and in the case of experienced investigators, Type 2 awards—these variables are assigned the means of R01 awardees (significantly higher for most measures according to Tables F and G and Figs B and C in S1 File). In the full sample, an additional 57% (3 ppt) of the predicted black/white funding gap is explained when the model is evaluated at the means of publications, bibliometrics and Type 2 awards for R01 awardees (Fig 4). We tested whether the difference between the white award probability and the black counterfactual award probability was statistically significant and failed to reject this hypothesis (p = .25). Providing the same average publications, bibliometrics, and Type 2 awards as the average R01 awardee essentially closes the black/white funding gap.

We conducted the same thought experiment for new investigators, and the gap nearly closes: 98% (4.2 ppt) of the predicted funding gap is explained by race/ethnicity differences in publications and associated bibliometrics, and that gap was not significantly different from zero (p = .94). For the experienced investigator sample, 36% (3 ppt) of the predicted black/white funding gap is explained by race/ethnicity differences in publications, bibliometrics, and Type 2 awards, but the gap was not significant (p = .22). In all cases, the difference between the white and black investigators’ probability of R01 awards evaluated at the means of R01 awardees are not significantly different. It is important to note that in the case of new and experienced investigators, the confidence intervals are wide given the smaller sample size. However, our sample contains almost all of the black investigators that applied for funding from 2003 to 2006.

Our previous study found that proposals that were resubmitted were more likely to be funded and that black investigators were less likely to resubmit proposals [2]. We estimated models that included controls for resubmitting proposals and evaluated the counterfactual predicted probability for black investigators at the mean characteristics of R01 awardees for publications and resubmissions (Fig C in S1 File). In the full sample and for new investigators, the predicted probability of black investigator R01 awards evaluated at the mean characteristics of R01 awardees exceeds that of white investigators. The predicted probabilities for white and black counterfactual experienced investigators are virtually identical.

### Identifying career divergence

**Fig 5:**
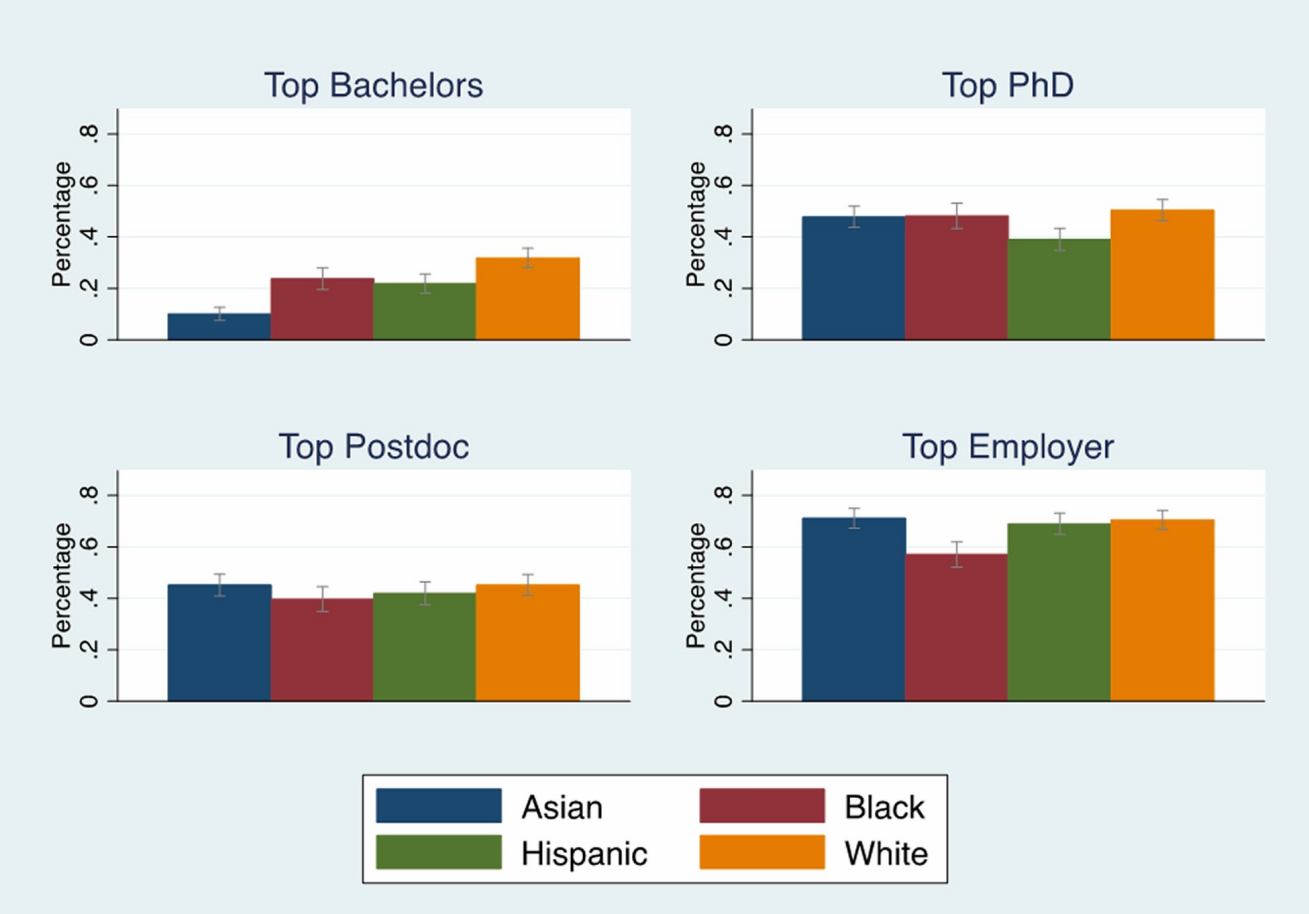
Trained/Employed at top 100 NIH funded institutions by race. Race/ethnicity differences in training and employment at the top 100 NIH funded institutions with 95% confidence intervals. Source: NIH IMPAC II, National Science Foundation Doctoral Record File, American Association of Medical Colleges faculty roster, select NIH Biosketches, Web of Science. N = 2,397.

We examined whether an applicant attended a top 100 NIH funded bachelor’s, doctoral and postdoctoral institution as well as a top 100 NIH ranked employer. Fig 5 (Tables F and G in S1 File) show that black investigators were less likely than white investigators to attend top bachelor’s institutions but equally likely to attend top doctoral institutions. Black investigators were significantly less likely (p < .001) to be employed at top 100 NIH funded institutions. Not having access to top bachelor’s programs does not seem to have disadvantaged black researchers at the PhD and postdoctoral stage. However, black investigators were less likely to be at top NIH funded employers.

**Fig 6:**
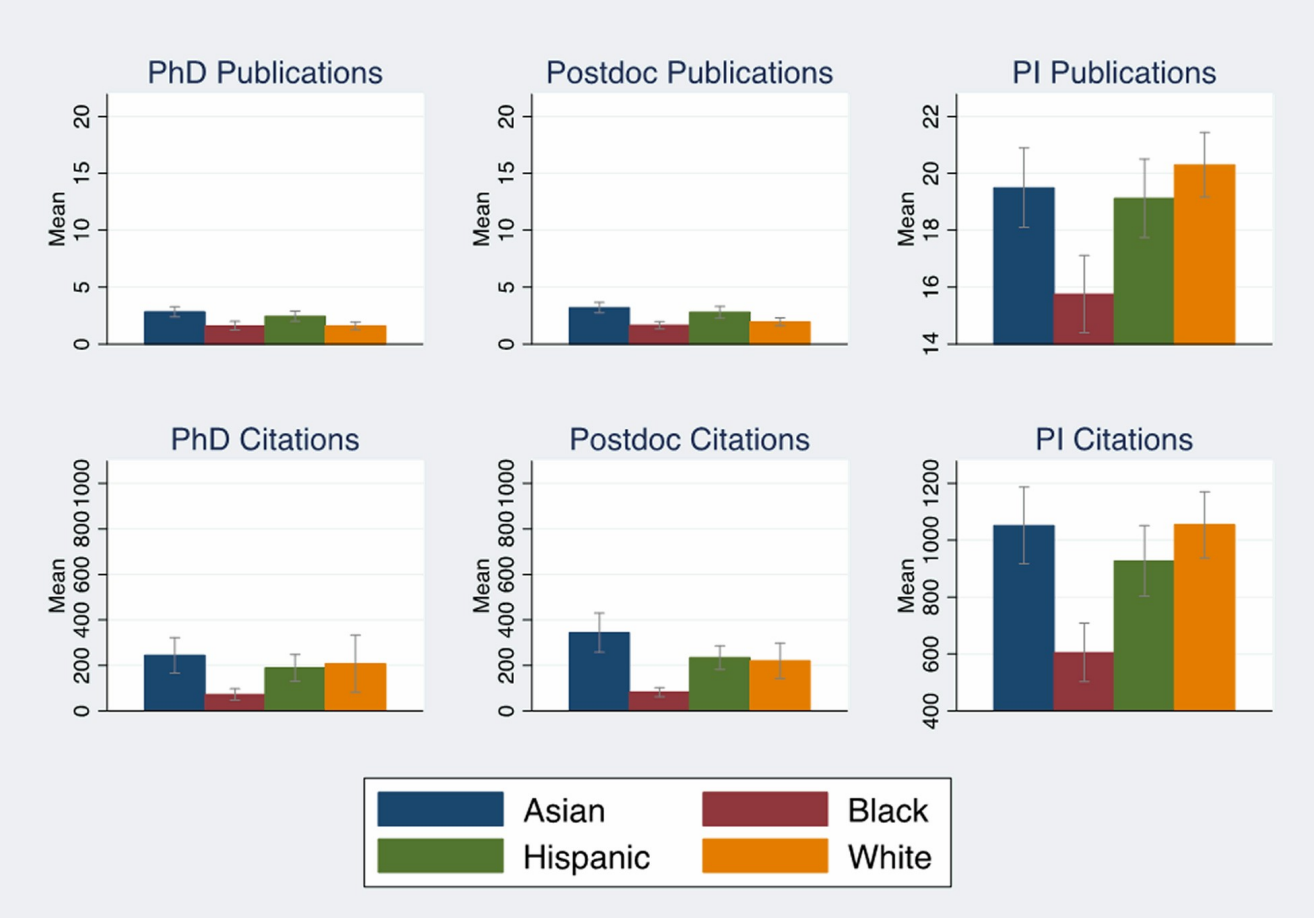
Average productivity by career stage, race, experienced investigators. Average publications and citations by career stage and race for experienced investigators with 95% confidence intervals. Source: NIH IMPAC II, National Science Foundation Doctoral Record File, American Association of Medical Colleges faculty roster, select NIH Biosketches, Web of Science. N = 1,165.

We examined differences in publications and citations by career stage as well in Fig 6 and Tables F and G in S1 File. Although black and white investigators published the same numbers of papers (approximately 2.5) during their PhD and postdoc years, papers from black investigators are cited less than half as much (p < .001) as papers by other race/ethnicity groups. Tables F and G in S1 File indicate that only publications during the postdoc are significantly lower for black new investigator awardees (p < .05) compared to white awardees. However, average black publications and citations at all career stages are significantly lower for new investigator non-awardees (p < .001), and black publications at the principal investigator stage are lower for non-awardee experienced investigators (p < .001) as are citations during the postdoc and principal investigator stage (p < .05, p < .001 respectively). This suggests that careers begin to diverge at the doctoral stage for new investigators and at the principal investigator stage for experienced investigators.

### Probability of receiving a priority score

Our previous analysis indicated that the probability of receiving a priority score on grant applications differed significantly by race/ethnicity. This is seen for the full sample, as well as for new and experienced investigators (Table B in S1 File). In Table 5, we demonstrated the effect of dropping unscored applications for full sample and new and experienced investigators. The black/white funding gap falls by half for both new and experienced investigators and is never statistically significant. We considered whether our new bibliometric measures and the full model could explain the black/white difference in receiving a priority score on a grant application in the last three columns of Table 4. Black investigators are -12.3 ppt (p < .001) less likely than whites to receive a priority score in the full sample, a much larger difference than the resulting funding gap of -6.9 ppt (Table 1). This gap was largely being driven by new investigators who were -11.7 ppt (p < .05) less likely to receive a score. Black experienced investigators were 10 ppt less likely to receive a score than whites, but this difference is not statistically significant at conventional levels (p = .11).

**Table 5:** Estimates of race/ethnicity differences in NIH funding and the probability of receiving a score.

		Drop Unscored		Probability of Score		
	Full	New	Experienced	Full	New	Experienced
VARIABLES	Model	Investigators	Investigators	Model	Investigators	Investigators
						
Asian	-0.007	-0.027	0.029	-0.034	0.051	-0.107*
	[0.041]	[0.064]	[0.054]	[0.031]	[0.047]	[0.042]
Black	-0.047	-0.017	-0.052	-0.123***	-0.117*	-0.104
	[0.045]	[0.069]	[0.064]	[0.032]	[0.043]	[0.050]
Hispanic	-0.014	-0.022	-0.010	-0.004	0.005	0.010
	[0.040]	[0.065]	[0.051]	[0.032]	[0.045]	[0.043]
Observations	2,397	1,232	1,162	2,397	1,232	1,162

Robust standard errors in brackets.

*** p<0.001

** p<0.01

* p<0.05.

Source: NIH IMPAC II, National Science Foundation Doctoral Record File, American Association of Medical Colleges faculty roster, select NIH Biosketches, Web of Science. All estimates use the full model described in Table A in S1 File.

## Discussion

This analysis has re-examined the black/white funding gap for NIH R01 awards identified in Ginther et al. [2] using detailed data on academic rank, scholarly awards, prior grant activity, training, and reported publications collected from Biosketches combined with bibliometric information from Web of Science. We found that academic rank, scholarly awards, prior non-NIH grants, and training had little explanatory power in our models. However our improved publication and bibliometric measures explained half of the black/white funding gap, and once the analysis was separated by investigator experience, the gap was no longer statistically significant for new investigators, and the gap narrowed and became less significant for experienced investigators. We also found that publications cut the black/white funding gap for MD/PhDs. We conducted a counterfactual analysis designed to simulate improved publication outcomes for black investigators. Assuming this intervention increased black publication outcomes, results from the full sample indicated that assigning the average publications, bibliometrics, and Type 2 awards of R01 awardees to black investigators closed the black/white funding gap by nearly 60%, and it was no longer statistically significant. The same thought experiment showed that practically all of the new investigator black/white funding gap could be closed if black investigators were assigned the average productivity of R01 awardees, and over one-third of the experienced investigator gap could be closed. Adding resubmissions to the model closed the funding gap for the full sample.

Why did black investigators report fewer papers that were cited less frequently on average than whites? And why did this gap widen with investigator experience? Our data suggested that cumulative advantage plays a role [29]. Although black investigators published the same number of papers during their PhD and postdoctoral training, these papers received fewer citations. This implies that black investigators may not receive the same advice from mentors related to research topics and publication strategies as whites during training and at the beginning of their careers. These disadvantages appear to accumulate as the black/white publication and citation gaps widen when black researchers become principal investigators (especially for investigators who were not funded). Furthermore, black researchers had fewer coauthors and fewer coauthors publishing in the top quartile of their research fields, a result identified elsewhere in the literature [30]. This indicates that black investigators had smaller professional networks that may constrain their publication counts, citations, and research impact as well as their prospects for promotion [30]. Finally, black publication records may be affected by institutional pressure to provide more service and mentor more students in order to promote the diversity initiatives of their institutions overburdening black researchers with a form of “cultural taxation” [31, 32].

The black/white funding gaps for new and experienced investigators are intriguing and instructive. By including more information from the Biosketch, the potential role of bias in the review process appears to be diminished. When black new investigators have publication records similar to all new investigator R01 awardees, the model predicts that they are equally likely to be funded as whites, and the gap for experienced investigators and the full sample closes considerably (Fig 4, Fig C in S1 File). The new investigator model also indicates that publications, bibliometrics, and the funding rank of the employer act as positive signals of the new investigator’s future research potential. Once a researcher becomes an experienced investigator, these factors have less explanatory power as evidenced by the lack of statistical significance of these variables in the experienced investigator model. For experienced investigators, receiving a Type 2 R01 award significantly increases the probability of a new Type 1 R01 award being funded. However, black experienced investigators receive one fewer Type 2 awards (p < .01) than whites.

These results have refined our understanding of the complex factors contributing to race/ethnicity differences in NIH R01 awards. We set out to distinguish between competing explanations: application merit, investigator characteristics, and unidentified factors that operate during the peer review process, including bias. Our approach leaned heavily on controlling for refined and expanded characteristics of the applicants in order to control for potential omitted variable bias. We succeeded in showing that publications and associated bibliometrics explained a substantial portion of race/ethnicity differences in funding. However, just because we can “explain” the gap does not mean that the problem is solved. Bias is notoriously difficult to identify in observational studies, with direct evidence of bias being found in audit studies that have come to different conclusions with respect to the role of gender in science careers [33, 34]. A recent study found that reviewers were not biased against NIH proposals from researchers with “Black names” [35]. However, this is a very restrictive test of bias since a name is only one factor associated with the ways that race is embedded in a researcher’s career. In fact, our previous study found that there was no funding gap for US citizen Asian investigators, and the four percentage point Asian gap resulted from Asian, foreign-born investigators [2]. This was despite the fact that Asian investigators publish the same or more papers and have a larger sum of impact factors than white investigators (Fig 2, Tables F and G in S1 File). It is possible that the peer review process may reflect bias against training from outside of the US. In addition, we found that new investigators and experienced Asian investigators were significantly less likely to receive a score on their proposals after controlling for all of the covariates in the full model (Table 5). Thus, bias could still be operating in the review and funding process given these remaining unexplained differences. Finally, bias could operate through other pathways outside of peer review and could be a factor in the quality of postdoctoral and early-career mentoring, or it could limit the size of professional networks that we observed.

Using the publication process to identify that careers diverge by race/ethnicity starting as early as the postdoc and then widen over time also is an important finding. These findings suggest opportunities for intervention. NIH announced pilot modifications to the Biosketch that allow a narrative account of “up to five of their most significant contributions to science” [36]. These changes may “level the Biosketch playing field,” perhaps reducing the weight that an individual’s publications might play in the assessment of merit. In addition, efforts recently announced by NIH to enhance the diversity of the scientific workforce, including the National Research Mentoring Network, may bolster professional networks for researchers from underrepresented groups [37]. A randomized trial of mentoring in the economics profession showed that the mentoring treatment improved the number of publications, the quality of publications, and the number of federal research grants for junior female economists [28]. Mentoring interventions should focus on conveying tacit knowledge related to the choice of research topics, grantsmanship, and the publication process for new investigators. In addition, institutions might consider lightening the service load for experienced black investigators so that the diversity needs of institutions do not derail their scientific research.

Given the importance of submitting a successful R01 Type 2 proposal for subsequent R01 Type 1 funding for experienced investigators, an investigation of the determinants of Type 2 award funding as a function of publications and bibliometrics is warranted. Previous studies have found mid-career researchers are disadvantaged [26] and that women are significantly less likely to receive Type 2 awards [38, 39]. Our data indicated blacks, Asians, and Hispanics had fewer Type 2 awards than whites. However, our data did not have the requisite information on Type 2 applications needed to probe these differences further. Although a recent study found no race/ethnicity differences in R01 awards after controlling for criterion and impact scores, their analysis did not model how race affected the decision to provide an overall impact score [7]. We could not fully probe this decision because the application review process employed during our study period is no longer in use at NIH. Under the current NIH review process [40], all applications are given an initial set of criterion scores ranging from 1 to 9 (best to worst) on the approach, significance, investigator, innovation and environment. The applications with the best criterion scores are “fully discussed” and then given an “overall impact” score. Although the “overall impact” score is not an aggregation of the criterion scores, there is significant correlation [41]. The WGDBRW report examined race/ethnicity differences in overall impact scores (for grants that received scores) and showed that blacks received slightly worse impact scores (1.2 points higher) than whites where scores are measured on the 10–90 point scale [9]. Considering that significant unexplained differences involve the score-no score decision, such analyses should be extended to preliminary criterion scores for applications that do not receive impact scores.

Our results also have emphasized the importance of using more robust measures of publications from the Biosketch to examine the determinants of R01 funding. Efforts such as ORCID [42] and Clarivate Analytics ResearcherID [43], which link researchers to their publications, and SciENCV [44], which creates structured Biosketch data, will facilitate any future analysis of the relationship between academic productivity and the probability of an application receiving a score. This suggests that combining the current NIH peer review system with covariates that explain the black/white funding gap identified in this and our previous analysis will likely yield insights about the factors associated with black/white differences in the probability of receiving an overall impact score and more explicitly inform opportunities for intervention.

## Supporting information

S1 File Materials and methods appendix. This appendix describes the processes used to select the sample used in the study, develop the data collection instrument, conduct data entry using IMPAC II application images for the sample, and ensure the accuracy of the data through standardized data cleaning and quality assurances procedures. **Fig A. Probability of R01 Award 2003–2006.** A comparison of R01 award probability by race/ethnicity, full sample from Ginther et al. [1] and subsample used in this analysis with 95% confidence intervals. Source: NIH IMPAC II, National Science Foundation Doctoral Record File, American Association of Medical Colleges faculty roster, select NIH Biosketches, Web of Science. Full Sample N = 53,454. Subsample N = 2,397. **Table A. Variables Used in Academic Rank, Prior Grants, Scholarly Awards, Publications Models.** Table A lists the variables used in models that include academic rank, prior grants, scholarly awards, publications and associated bibliometrics. **Fig B. Average Number of Type 2 Awards by Race.** Average number of Type 2 R01 Awards for Experienced Investigators by race/ethnicity, 2003–2006 with 95% confidence intervals. Source: NIH IMPAC II, National Science Foundation Doctoral Record File, American Association of Medical Colleges faculty roster, select NIH Biosketches, Web of Science. N = 2,397. **Fig C. Predicted Probability of R01 Award—Resubmission Model.** Predicted probability of NIH R01 awards by race and investigator experience including controls for proposal resubmissions with 95% confidence intervals. Source: NIH IMPAC II, National Science Foundation Doctoral Record File, American Association of Medical Colleges faculty roster, select NIH Biosketches, Web of Science. N = 2,397. **Table B. Variables Used in Training Models.** Table B lists the variables used in models that include characteristics related to undergraduate institutions, predoctoral activities, PhD institution, postdoctoral appointments and fellowships. **Table C. Probit Estimates of NIH R01 Award Controlling for Training Characteristics.** Table C shows probit estimates of the effect of training variables on race/ethnicity differences in NIH awards.**Table D. Contribution of Covariates to Explained Portion of the Black NIH Funding Gap.** Table D shows linear probability estimates of the percentage of race/ethnicity gap in NIH funding explained by covariates. **Table E. Counts of Applications and Awards by Race and Investigator Status.** Table E shows the counts of applications, scored applications and awards by race/ethnicity and investigator experience. Experienced investigators are significantly more likely to receive scores and funding. **Table F. Average of Productivity Measures by Race/Ethnicity for New Investigators, NIH R01 Awardees and Non-awardees.** Table F lists average productivity (publication and bibliometric) measures by race/ethnicity, R01 award status and investigator experience. Regardless of award status, black investigators publish fewer papers, are cited less often, and publish in lower impact journals than whites. **Table G. Average of Productivity Measures by Race/Ethnicity for Experienced Investigators, NIH R01 Awardees and Non-awardees.** Table G lists average productivity (publication and bibliometric) measures by race/ethnicity, R01 award status and investigator experience. Regardless of award status, black investigators publish fewer papers, are cited less often, and publish in lower impact journals than whites.
(DOCX)
