# Supplementary material for: Publications as predictors of racial and ethnic differences in NIH research awards
Source: PLoS One. 2018 Nov 14;13(11):e0205929. doi: 10.1371/journal.pone.0205929 (PMC6235266; doi:10.1371/journal.pone.0205929)
Supplement: S1 File — This appendix describes the processes used to select the sample used in the study, develop the data collection instrument, conduct data entry using IMPAC II application images for the sample, and ensure the accuracy of the data through standardized data cleaning and quality assurances procedures. Fig A. Probability of R01 Award 2003–2006. A comparison of R01 award probability by race/ethnicity, full sample from Ginther et al. [1] and subsample used in this analysis with 95% confidence intervals. Source: NIH IMPAC II, National Science Foundation Doctoral Record File, American Association of Medical Colleges faculty roster, select NIH Biosketches, Web of Science. Full Sample N = 53,454. Subsample N = 2,397. Table A. Variables Used in Academic Rank, Prior Grants, Scholarly Awards, Publications Models. Table A lists the variables used in models that include academic rank, prior grants, scholarly awards, publications and associated bibliometrics. Fig B. Average Number of Type 2 Awards by Race. Average number of Type 2 R01 Awards for Experienced Investigators by race/ethnicity, 2003–2006 with 95% confidence intervals. Source: NIH IMPAC II, National Science Foundation Doctoral Record File, American Association of Medical Colleges faculty roster, select NIH Biosketches, Web of Science. N = 2,397. Fig C. Predicted Probability of R01 Award—Resubmission Model. Predicted probability of NIH R01 awards by race and investigator experience including controls for proposal resubmissions with 95% confidence intervals. Source: NIH IMPAC II, National Science Foundation Doctoral Record File, American Association of Medical Colleges faculty roster, select NIH Biosketches, Web of Science. N = 2,397. Table B. Variables Used in Training Models. Table B lists the variables used in models that include characteristics related to undergraduate institutions, predoctoral activities, PhD institution, postdoctoral appointments and fellowships. Table C. Probit Estimates of NIH R01 Award Controlling f [file pone.0205929.s001.docx]

Supplementary Materials for

Publications as Predictors of Racial and Ethnic Differences in

NIH Research Awards

Donna K. Ginther^[[1]](#footnote-1)^*, Jodi Basner^2^, Unni Jensen^[[2]](#footnote-2)^, Joshua Schnell2, Raynard Kington^[[3]](#footnote-3)^,

Walter T. Schaffer^[[4]](#footnote-4)^

Correspondence to: dginther@ku.edu

**This PDF file includes:**

Materials and Methods

Supplementary Text

A-C Figs

A-G Tables

References [1-9]

**Materials and methods**

This appendix describes the processes used to select the sample used in the study, develop the data collection instrument, conduct data entry using IMPAC II application image files for the sample, and ensure the accuracy of the data through standardized data cleaning and quality assurance procedures. It also describes the methods for binning and coding the extracted data to further categorize it and prepare it for analysis. In addition, we provide supplemental tables and graphs discussed in the paper.

The paper uses a sample of 2,397 observations from Ginther et al. [1], and several variables used in this analysis were developed in the previous study. Details on the data and variable construction in that study can be found by referring to the online supplemental material [2].

**Details of data linkage and sample selection**

To perform our new research, we drew a random sample of applications within the four self-reported racial groups of Asian, black, Hispanic, and white from the 83,188 applications for NIH R01 grants described by Ginther et al. [1]. The applications were collected for Fiscal Years (FY) 2003 to 2006 in order to have information from the Biosketch images available in the IMPAC II data set. We selected 600 applications within each race group for a total of 2400 observations. Applications were selected at random, without replacement, and multiple applications from the same individual were allowed. The maximum number of applications for an individual in the final sample was 11; in the candidate set, it was 38. The *sampling rate* used for a given race equals the number in the sample for a given race divided by the number in the candidate set for a given race. Since blacks and Hispanics were under-represented among NIH R01 applicants, we oversampled these groups relative to whites and Asians. The resulting sample rates are:

- Asian: 7% (600/8977)
- Black: 82% (600/729)
- Hispanic: 34% (600/1732)
- White: 1.6% (600/35825)

The alternative, making the sample *rate* constant, was not viable because making it low (e.g., for the black population) would not provide enough statistical power to measure the effects of interest, and making it high would require a level of effort beyond the scope of the project.

S1 Fig compares the award probabilities for the full candidate sample from Ginther et al. [1] for grants from FY 2003-2006 and the random subsample used in the analysis. R01 award probabilities for the random sample employed in this study were comparable to those in the candidate population, although the confidence intervals on award probability were larger for the Asians, Hispanics, and whites, as expected. Award probabilities are slightly higher for Asians, Hispanics, and whites in the subsample compared to the full sample. Award probabilities for blacks are the same, perhaps reflecting the fact that we over-sampled black applicants.

Since the outcome variables in our race-stratified random sample reflect the properties of the full sample, and our analysis is based on regression estimates, we do not weight the data. When stratification is based on an exogenous variable, in this case, race, the unweighted estimator is consistent and asymptotically normal [3].

**Data from NIH biographical sketches**

A data model was designed to capture key variables from the biographical sketch associated with each application in the IMPAC II database into an Excel spreadsheet for the 2,400 applications that comprised the sample. After development and testing by four Clarivate Analytics Data Entry Specialists using a core set of applications, the data model was refined by the Project Manager and Associate Consultants and implemented by a team of Data Entry Specialists.

Data were collected from the applications' Face Pages, the Biographical Sketches of Principal Investigators, and from Google searches, when information was not available in the application (e.g., location of an institution where an applicant had previously trained). Data entered as the result of an online search were highlighted in red so that an additional check could be made during the data cleaning and quality assurance processes.

The Excel spreadsheet included seven tabs that captured data on: A) Faculty rank of applicant at the time of application; B) Undergraduate degree(s); C) PhD degree(s); D) Postdoctoral research positions; E) Publications; F) Scholarly awards and advisory panel experience; G) Prior grants.

1. **Faculty rank and institutional affiliation**

Faculty Rank and institutional affiliation were entered manually from the Biosketch and then the accuracy of this information was checked by other members of the data entry team. The Lead Data Entry Specialist ran a query to verify Faculty Rank entries (i.e., position titles) against a list of valid titles and convert them to a numeric value (see Exhibit 1). In some cases the Faculty Rank entries were manually updated and edited; for example, when they included an area of specialization in addition to the position title (e.g., "Professor of Veterinary Medicine" was edited to "Professor"). These values were entered in a separate column in the spreadsheet.

**Exhibit 1. Faculty Rank Values**

1 = Associate Professor

2 = Assistant Professor

3 = Professor

4 = Research Assistant Professor

5 = Research Associate Professor

6 = Clinical Assistant Professor

7 = Clinical Associate Professor

8 = Other Research (e.g., Research Assistant, Senior Research Assistant, Research Associate, Staff Scientist, Staff Investigator, Scientist)

9 = Other Non-Research (e.g., President, Physician, Director)

Our data entry process also standardized the names of US institutions attended by the applicants against the Carnegie Classification of Institutions [4] and standardized the names of foreign institutions. This process was conducted for applicant organizations; undergraduate, graduate, and postdoctoral institutions; and private institutions at which postdoctoral work was completed. The standardized names were entered in a separate column and sent to a Principal Consultant.

We binned institutional information into the following categories: Historically Black Colleges and Universities (HBCU) [5], Carnegie Classification, Oberlin 50 status [6], Association of American Medical Colleges (AAMC) membership, Department of Education Integrated Postsecondary Education System (IPEDS) classification, National Research Council (NRC) ranking, private organization, or research institute.

1. **Undergraduate degrees**

We coded information on the year in which the undergraduate degree was received, the field of study, and whether the field of study is in the area of Science/Engineering (e.g., Biology, Health Sciences, Chemistry, Engineering); Social/Behavioral studies (e.g., Psychology, Sociology, Social Work); or Other (e.g., Education, Humanities, English). If no undergraduate degree was listed in the Biosketch, we controlled for undergraduate degree missing in the analysis. In the case of a double major, each was entered on a separate line. We also created dummy variables to indicate awards/honors at the undergraduate level; undergraduate research experience, if any; the year in which the research was completed; and advisor name(s). If more than one research experience was listed, the number of years of experience was entered as well. The remaining fields capture the undergraduate institution and related city, state, and country.

1. **PhD degrees**

Ginther et al. [1] had information from the Survey of Earned Doctorates on PhD degree characteristics for those who studied in the United States. We coded the year in which the PhD or PhD equivalent was received, PhD advisor(s) noted in the Biosketch, the name of the institution from which the PhD was received, and the city, state, and country of the applicant organization. PhD equivalents are shown in Exhibit 2.

Few applicant Biosketches provided the names of advisors at any educational level. We matched PI names to the ProQuest database to identify dissertation advisors. ProQuest is a database containing over 2 million doctoral dissertations and theses. Using ProQuest, the data analysts searched for applicants in the sample by last name, first name, PhD year, and PhD institution, using metadata from the dissertation record to collect advisor names where available. However, not all dissertations appeared in ProQuest, and some of those that did appear did not include the name of the doctoral advisor. Thus, we were not able to identify a sufficient number of PhD advisors to incorporate these data in the analysis.

| **Exhibit 2. PhD Equivalents**   - DMSC - DNS - DNSC - DPH - DPHI - DPHIL - DPHL - DRPH - DRSC - DSC - DSW - ED - EDD - PDFELLOW - PharmD - SCD - SciD - SD - DMEDSC |
| --- |

1. **Postdoctoral training**

Our 2,025 applicants listed 2,802 postdoctoral appointments. We collected information on each postdoctoral position listed on the Biosketch. If one doctoral degree was obtained by conducting research at more than one institution, only the first institution was listed. We coded information on the year the postdoc was completed, postdoctoral advisor(s) (if listed), the name of the awarding institution or organization, and the city, state, and country of the institution. If the application indicated that the postdoc was still in progress, the year of the application was entered.

Only 250 applicants listed their postdoctoral advisor. We performed Google searches and were able to identify a total of 527 postdoctoral advisors. These data were insufficient to analyze the impact of postdoctoral advisor on application success and consequently were not used in the study.

1. **Publications**

The Publications section had columns for placement of the application number and the list of peer-reviewed publications provided in the Biosketch. Items such as edited books, book chapters, and presentations were not collected.

Nearly 54,000 publications were identified from the Biosketches and linked to Medline and Web of Science. We retrieved bibliometric measures, including citations, from Web of Science. Unlike Ginther et al. [1] we could confirm these publications were written by the applicant. In Ginther et al. [1] we used a name-matching strategy to link publications to R01 applicants. Our decision rules resulted in undercounting publications (and as a result, the associated bibliometrics). The present study identified an average of 22.5 publications per application, whereas Ginther et al. [1] identified an average of 17.6 publications per application.

Using the years of PhD and postdoctoral study, we attributed publications (and associated bibliometrics) to the predoctoral/PhD training, postdoctoral training, and principal investigator career stages. When advisor names were available, the appearance of those names on publications was used to determine which of the applicants’ publications resulted from predoctoral and postdoctoral research. When advisor names were unavailable, dates of training were used.

Bibliometric measures were collected for each publication, including total citations, two-year citations, citations excluding self-citations, journal subject category, number of unique coauthors, number of unique last authors, number of first-authored and last-authored publications, and impact factor of the journal [7, 8].

These bibliometric measures were used to create field-normalized bibliometrics. Each field (measured by journal subject category) has different citation patterns and journals in the fields have different impact factors. For example, according the 2013 Journal Citation Reports [9], the top journal in Biochemistry and Molecular Biology has an impact factor of 33.12 and the top journal in Biological Psychology has an impact factor of 14.96. Thus if we treat each citation and impact factor the same without controlling for field, we may under (or over) estimate the impact of a particular publication. Therefore we used field-normalized measures based on the journal subject category of the publication. Citations to the publication over the first two years were compared to the reported number of citations to papers over the first two years in the same journal subject category. The publication was then assigned a field-normalized quartile rank in the publication distribution as available in Clarivate Analytics Journal Citation Reports. We identified the percentage of the applicant’s publications that were published in each quartile for the observed field. In addition, we calculated the percentage of papers that were uncited, the percentage of field-normalized papers in the top quartile of citations in that field, the sum of the impact factors for all of the applicant’s publications, and the citation-weighted sum of the impact factors where citations to a publication in a given journal are multiplied by the impact factor of the journal and these are summed across all publications.

We identified the three most common last authors and the three most common coauthors (who were not last authors) on the publications and developed field-normalized measures for publications on the Biosketch from these individuals. We expected that last-authored publications could be used as a proxy for PhD and postdoctoral advisor for individuals at those career stages, but only 56% of last-authored publications matched reported PhD and postdoctoral advisors. These data were viewed as unreliable measures of advisors. We used these data to examine whether collaborations with highly productive last authors and other coauthors were associated with R01 application success. We found that coauthors who were cited in the top quartile of their field were associated with R01 application success, but last authors were not.

We created several versions of these bibliometric measures and picked the model that fit the data best based on minimizing the Bayesian Information Criterion (BIC), the Akaike Information Criterion, and the percentage of correct predictions of the model. This analysis indicated that the natural log of the sum of the impact factors fit the data better than including categorical variables for the number of publications and citations (the approach used in Ginther et al. [1]). In addition, we included controls for the percentage of first-authored publications, the percentage of last-authored publications, the percentage of papers in the top quartile of the applicant’s most-frequent journal subject category (field-normalized measure), the percentage of uncited papers, and the percentage of the coauthors’ papers in the top quartile of the field.

1. **Scholarly awards**

Scholarly Awards include notable accomplishments listed under Awards and Honors in the Biosketch, as well as awards, training programs, and grants listed under Other Experience or Professional Memberships, Awards and Fellowships, and Memberships. Service on advisory and review panels, editorial boards, and leadership activities were also collected in this section. Honorary degrees do not count as awards, nor do research grants.

The raw data for Scholarly Awards were evaluated for coding during the data cleaning process. The first task was to determine whether the award or honor should be categorized into one of the following columns in the data collection instrument: Fellowship, Traineeship, Minority Component, Loan Repayment Program, Advisory Panel Experience (in which case the Advisory Panel Type column was also completed), Editorial Board Experience, Editor Experience, or Leadership. The Leadership category was intended for awards at the national level that are not paid positions. Google searches were undertaken when it was not clear which of these categories, if any, was the best match. If the applicant organization listed activities that did not fit the parameters established for Scholarly Awards, they remained in the original Scholarly Awards column of the spreadsheet.

Once the raw data for Advisory Panels was identified, it was coded into six categories, as shown in Exhibit 3.

**Exhibit 3. Advisory Panel Categories**

1 = NIH Special Emphasis Panel
2 = NIH Standing Review Panel
3 = National Advisory Council/Board
4 = Program Advisory Committee/Council
5 = Journal Reviewer (not an Editor)
6 = Other Grant Review (non-NIH review)

Special Emphasis Panels were explicitly noted by the applicant as "SEP", "special reviewer," or "special panel." Examples include:

- 2003 NIH ZRG SNEM-2 50 Special Emphasis Panel "Pathways Linking Education to Health”
- Special Review Committee for Program Project, NIH National Heart, Lung and Blood Institute, 2004
- Special Emphasis Panel, National Institute on Drug Abuse, July 8, 2003

Standing Review Panels are long-term NIH review panels. They were usually indicated by the phrases “ad hoc reviewer,” or “standing review panel.” Examples include:

- 2002 Ad Hoc Reviewer, NIH; Fogarty International Research Collaboration Award
- 2003-2007 Study Section Member, National Institutes of Health/Fogarty International Research Collaboration Awards, Bethesda, MD
- 2000-Present NIDA-BGES Scientific Review Committee

National Advisory Councils/Boards are advisory panels with national scope, such as a committee formed by a national professional society or organization or non-NIH federal advisory committees. Examples include:

- Board Member, Society for the Analysis of African-American Public Health Issues
- Co-Chair, U.S. Public Health Services, Office on Women's Health, Minority Women's Health Panel of Experts
- 1997-1998 American Association of Colleges of Nursing, Commission on Collegiate Nursing Education, Standards Committee.

Program Advisory Councils/Boards are advisory panels for a specific program or a localized (e.g., state) scope. Examples include:

- Member of the ADAP Advisory Council to the Texas Board of Health
- Member, Louisiana Substance Abuse Prevention Programs and Needs Assessment Task Force, Office of Alcohol and Drug Abuse, Department of Health and Hospitals, 1997-1998
- Co-Chair, APA Division 44, SPSLGM, Committee of Ethnic Minority Affairs

A Journal Reviewer is an individual who reviewed papers for a journal, but was not on an Editorial Board and was not an Editor (that data was collected in separate columns, as noted above). Examples include:

- 2001 Ad Hoc Reviewer, J. Cellular & Molecular Biology
- 2003 Ad Hoc Reviewer, J. Clinical Anatomy
- 2003 Ad Hoc Reviewer, Cultural Diversity and Ethnic Minority Psychology

Other Grant Reviews are reviews of applications for funding from non-NIH sources, such as the National Science Foundation. Examples include:

- 1999-2001 Member, American Heart Association/Cellular Physiology and Pharmacology Study Group C Reviewer
- 2003 Michigan Women's Foundation Social Impact Grants, Grant Review
- 1992 Grant Reviewer for USAID

Any identified honors received at the undergraduate level, any undergraduate research experience, and the number of years of undergraduate research listed under honors were assigned to undergraduate research/honors categories.

In some cases, applicants presented prior grants, patent data, or positions under the Awards/Honors category in their Biosketches. We categorized these data under the appropriate headings instead of Honors/Awards.

1. **Prior grants**

In Ginther et al. [1] we identified all prior NIH grants, however, that study had no information on prior grants from other organizations. Using Biosketch data, we entered and categorized all completed and ongoing grants awarded to the applicant that were listed, as long as the roles of the applicant (e.g., PI, Co-PI) were provided. Pending grants were not entered. The Program Name field was completed if the grant was awarded as part of a specific program, such as an NIH FIRST Research Award or a Higher Education Emerging Scholars Program. The Funding Organization column lists the specific funding agency or organization, such as NIH or the American Cancer Society. The Grant Role Type indicates the role of the applicant in each grant listed, including Principal Investigator, Co-Principal Investigator, Director, or Other. If an applicant had more than one role on a grant, each role was listed separately. The Applicant Career Stage field indicates whether work on the grant took place prior to or after receipt of the PhD. The direct costs of the grant were recorded if reported on the Biosketch. We also identified any minority-focused programs such as "MARC" or "Minority Access." These grants were sometimes listed in the Awards/Honors section of the Biosketch.

The raw data were categorized according to the taxonomy in Exhibit 4. Initially, non-Federal organizations were assigned values manually. As the data analysts identified and categorized the non-Federal organizations, they were able to automate the process. When necessary, Google searches were conducted to identify information on unfamiliar foundations or nonprofit organizations to ensure that they were assigned the correct values. Analysts conducted queries to ensure that grants with minority components (i.e., directed toward minority populations) were identified and entered in the spreadsheet.

**Exhibit 4. Funding Organization Values**

1 = NIH

2 = Other Federal Agency
3 = Foundation/Nonprofit Disease Focused
4 = Foundation/Nonprofit Broad Research

5 = University or Research Institute
6 = State Government
7 = Private Industry
8 = International
9 = Unknown

**Data cleaning and quality assurance**

Following data entry, the data were cleaned and evaluated for quality. "Cleaning" refers to the identification and elimination of errors that would prevent the data from being properly uploaded to the database. (An automated data loading program was created to import the data from the data collection instrument created in Excel to a SQL server database.) The first steps in cleaning were to import the data into Microsoft Access and run a query that performed automated corrections, such as fixing invalid formats and identifying erroneous grant numbers. A query was run to ensure that degree types were categorized correctly as Science/Engineering, Social/Behavioral, or Other for Field of Study. Applicants without a PhD or PhD equivalent were deleted from the tabs (yet maintained in the sample) by running a query designed for that purpose. Additional manual cleaning processes required changing text to numerals in the funding amount and year fields where necessary, correcting other invalid values, and eliminating extra characters.

Once the data were cleaned, a comprehensive Quality Assurance (QA) process was conducted. Every field was checked against the original application for approximately 10 percent of the applications (there were 55 items per application). This ensured that the number of data entry errors did not exceed the allowable threshold. In fact, the Data Entry Inter-Coder Reliability was 98 percent. All identified errors were corrected. Missing data points under any tab (e.g., city, state) were completed, which in some cases required Google searches. Entries marked in red (indicating searches by other Data Entry Specialists) were verified with a second search. Data also were scanned for obvious errors and red flags. Comments entered in the Notes column by the Data Entry Specialists were read and addressed. If errors or anomalies were identified during any of these processes, the Lead Data Entry Specialist corrected the data.

**Supplementary text**

**A Fig** shows a comparison of R01 Award Probability by race/ethnicity, both the full Sample from Ginther et al. [1] and the subsample used in this analysis with 95% confidence intervals.

**B Fig** shows the average number of Type 2 R01 awards received by experienced investigators by race/ethnicity. Blacks have received less than one Type 2 award on average whereas whites average two awards.

**C Fig** shows the predicted probability of NIH funding for white and black investigators evaluated at the average characteristics of R01 awardees for publications, bibliometrics, and resubmissions. The combination of these characteristics increases the black counterfactual predicted award probability so that it exceeds white predicted award probability in the full sample and for new investigators.

**A Table** lists the variables used in models that include academic rank, prior grants, scholarly awards, publications and associated bibliometrics.

**B Table** lists the variables used in models that include characteristics related to undergraduate institutions, predoctoral activities, PhD institution, postdoctoral appointments and fellowships.

**C Table** shows probit estimates of the effect of training variables on race/ethnicity differences in NIH awards.

**D Table** shows linear probability estimates of the percentage of race/ethnicity gap in NIH funding explained by covariates.

**E Table** shows the counts of applications, scored applications and awards by race/ethnicity and investigator experience. Experienced investigators are significantly more likely to receive scores and funding.

**F** and **G Tables** list average productivity (publication and bibliometric) measures by race/ethnicity, R01 award status and investigator experience. Regardless of award status, blacks publish fewer papers, are cited less often, and publish in lower impact journals than whites.

**A Fig.**

**B Fig.**

**C Fig.**

**A Table. Variables Used in Academic Rank, Prior Grants, Scholarly Awards, Publications Models**

|  | **Main** | **Rank** | **Grants** | **Scholarly Honors** | **Publications** | **Field-Normed Pubs** | **Coauthor Pubs** | **Journal Field** | **Full Model** |
| --- | --- | --- | --- | --- | --- | --- | --- | --- | --- |
| **Demographic Variables** |  |  |  |  |  |  |  |  |  |
| Race/Ethnicity | X | X | X | X | X | X | X | X | X |
| Gender | X | X | X | X | X | X | X | X | X |
| Age | X | X | X | X | X | X | X | X | X |
| Foreign PhD | X | X | X | X | X | X | X | X | X |
| Foreign PhD-missing | X | X | X | X | X | X | X | X | X |
| Fiscal Year Indicators (03, 04, 05) | X | X | X | X | X | X | X | X | X |
| **Covariates** |  |  |  |  |  |  |  |  |  |
| Employer NIH Funding Rank: |  |  |  |  |  |  |  |  | X |
| (Top 30, 31-100, 100-200) b |  |  |  |  |  |  |  |  | X |
| Prior NIH Grants |  |  |  |  |  |  |  |  | X |
| NIH Review Committee |  |  |  |  |  |  |  |  | X |
| Human Subjects |  |  |  |  |  |  |  |  | X |
| NIH Institute Code: |  |  |  |  |  |  |  |  | X |
| (8 Indicators for IC receiving proposal) |  |  |  |  |  |  |  |  | X |
| Grant Resubmitted (1, 2+) |  |  |  |  |  |  |  |  | X |
| **Academic Rank** |  |  |  |  |  |  |  |  |  |
| Assistant Professor |  | X |  |  |  |  |  |  |  |
| Associate Professor |  | X |  |  |  |  |  |  |  |
| Full Professor |  | X |  |  |  |  |  |  |  |
| Clinical Professor |  | X |  |  |  |  |  |  |  |
| Research Professor |  | X |  |  |  |  |  |  |  |
| **Prior Grants Counts** |  |  |  |  |  |  |  |  |  |
| Prior NIH Grants--Biosketch |  |  | X |  |  |  |  |  |  |
| Non-NIH Grants |  |  | X |  |  |  |  |  |  |
| Federal Grants (e.g. NSF) |  |  | X |  |  |  |  |  |  |
| Disease Grants |  |  | X |  |  |  |  |  |  |
| Foundation Grants |  |  | X |  |  |  |  |  |  |
| Research Institute Grants |  |  | X |  |  |  |  |  |  |
| State Grants |  |  | X |  |  |  |  |  |  |
| Industry Grants |  |  | X |  |  |  |  |  |  |
| Number of Grants as PI |  |  | X |  |  |  |  |  |  |
| Number of Grants as Co-I |  |  | X |  |  |  |  |  | X |
| Number of Grants as Director |  |  | X |  |  |  |  |  |  |
| **Scholarly Honors/Awards** |  |  |  |  |  |  |  |  |  |
| Fellowships |  |  |  | X |  |  |  |  |  |
| Traineeships |  |  |  | X |  |  |  |  |  |
| Minority-related awards |  |  |  | X |  |  |  |  |  |
| Loan Repayment awards |  |  |  | X |  |  |  |  |  |
| NIH Special Emphasis Panel |  |  |  | X |  |  |  |  |  |
| NIH Standing Review Committee |  |  |  | X |  |  |  |  |  |
| National Advisory Panel |  |  |  | X |  |  |  |  |  |
| Program Committee Organizer |  |  |  | X |  |  |  |  |  |
| Non-NIH Panel Member |  |  |  | X |  |  |  |  |  |
| Journal Reviewer |  |  |  | X |  |  |  |  |  |
| **Publications** |  |  |  |  |  |  |  |  |  |
| Zero or Missing Publications |  |  |  |  | X | X | X | X | X |
| Natural Log of Sum of Impact Factors |  |  |  |  | X | X | X | X | X |
| Percentage of First Authored Papers |  |  |  |  | X | X | X | X | X |
| Percentage of Last Authored Papers |  |  |  |  | X | X | X | X | X |
| Percentage of Uncited Papers |  |  |  |  |  | X | X | X | X |
| Percentage in of Papers in Top Quartile of Field (Field Normalized) |  |  |  |  |  | X | X | X | X |
| Percentage of Coauthors papers in Top Quartile of field (Field Normalized) |  |  |  |  |  |  | X | X | X |
| 19 Indicators for major publication field |  |  |  |  |  |  |  | X |  |
| **Experienced Investigators** |  |  |  |  |  |  |  |  |  |
| Type 2 R01 Award Indicator |  |  |  |  |  |  |  |  | X |

**B Table. Variables Used in Training Models**

|  | **Main** | **Undergrad** | **Predoc** | **PhD** | **Postdoc** | **NIH PD** | **Fellowships** | **All Training** | **US Citizens, NIH** |
| --- | --- | --- | --- | --- | --- | --- | --- | --- | --- |
| **Demographic Variables** |  |  |  |  |  |  |  |  |  |
| Race/Ethnicity | X | X | X | X | X | X | X | X | X |
| Gender | X | X | X | X | X | X | X | X | X |
| Foreign PhD | X | X | X | X | X | X | X | X | X |
| Foreign PhD-missing | X | X | X | X | X | X | X | X | X |
| Fiscal Year Indicators (03, 04, 05) | X | X | X | X | X | X | X | X | X |
| **Undergraduate Variables** |  |  |  |  |  |  |  |  |  |
| NIH Funding Rank of BA Institution: |  | X |  |  |  |  |  | X |  |
| (Top 30, 31-100, 100-200) ^b^ |  | X |  |  |  |  |  | X |  |
| BA Oberlin 50 |  | X |  |  |  |  |  | X |  |
| BA Foreign |  | X |  |  |  |  |  | X |  |
| Undergrad Honors |  | X |  |  |  |  |  | X |  |
| **Predoctoral Variables** |  |  |  |  |  |  |  |  |  |
| NIH F31 |  |  | X |  |  |  |  | X | X |
| NIH Diversity F31 |  |  | X |  |  |  |  | X | X |
| Number of T32 Predocs (1,2,3+) |  |  | X |  |  |  |  | X | X |
| **PhD Variables** |  |  |  |  |  |  |  |  |  |
| Foreign PhD |  |  |  | X |  |  |  | X |  |
| NIH Funding Rank of PhD Institution: |  |  |  | X |  |  |  | X |  |
| (Top 30, 31-100, 100-200) ^b^ |  |  |  | X |  |  |  | X |  |
| PhD from Historically Black College |  |  |  | X |  |  |  | X |  |
| **Postdoctoral Variables** |  |  |  |  |  |  |  |  |  |
| Number of Postdocs (1, 2, 3+) |  |  |  |  | X | X | X | X |  |
| Foreign Postdoc |  |  |  |  | X | X | X | X |  |
| NIH Funding Rank of 1st Postdoc Institution: |  |  |  |  | X | X | X | X |  |
| (Top 30, 31-100, 100-200) ^b^ |  |  |  |  |  |  |  |  |  |
| NIH F32 Fellowship |  |  |  |  |  | X | X | X | X |
| Other F Fellowship |  |  |  |  |  | X | X | X | X |
| K 14 Fellowship |  |  |  |  |  | X | X | X | X |
| Other K Fellowship |  |  |  |  |  | X | X | X | X |
| Number of T32 Postdocs (1, 2) |  |  |  |  |  | X | X | X | X |
| **Fellowship Variables** |  |  |  |  |  |  |  |  |  |
| Foundation Fellowship |  |  |  |  |  |  | X | X |  |
| Foreign Fellowship |  |  |  |  |  |  | X | X |  |
| Disease Fellowship |  |  |  |  |  |  | X | X |  |
| University Fellowship |  |  |  |  |  |  | X | X |  |
| Professional Society Fellowship |  |  |  |  |  |  | X | X |  |
| Minority Non-Federal Fellowship |  |  |  |  |  |  | X | X |  |
| Minority Federal Fellowship |  |  |  |  |  |  | X | X | X |

**C Table. Probit Estimates of NIH R01 Award Controlling for Training Characteristics**

|  | | Main | Undergraduate | Pre Doctoral | PhD Characteristics | Post Doctoral | NIH Postdoctoral | Non-NIH Fellowships | All Training Variables | US Citizens NIH Training |
| --- | --- | --- | --- | --- | --- | --- | --- | --- | --- | --- |
| VARIABLES | |  |  |  |  |  |  |  |  |  |
|  | |  |  |  |  |  |  |  |  |  |
| Asian | | -0.044* | -0.028 | -0.028 | -0.045* | -0.041 | -0.037 | -0.039 | -0.020 | -0.012 |
|  | | [0.022] | [0.025] | [0.025] | [0.022] | [0.022] | [0.023] | [0.022] | [0.025] | [0.047] |
| Black | | -0.134*** | -0.131*** | -0.130*** | -0.134*** | -0.128*** | -0.126*** | -0.127*** | -0.123*** | -0.119*** |
|  | | [0.020] | [0.020] | [0.020] | [0.020] | [0.020] | [0.020] | [0.021] | [0.021] | [0.027] |
| Hispanic | | -0.039 | -0.034 | -0.031 | -0.037 | -0.038 | -0.038 | -0.038 | -0.031 | -0.033 |
|  | | [0.022] | [0.023] | [0.023] | [0.023] | [0.023] | [0.023] | [0.023] | [0.023] | [0.031] |
| Had three or more postdocs | |  |  |  |  | -0.100* | -0.101* | -0.097* | -0.099* |  |
|  | |  |  |  |  | [0.048] | [0.048] | [0.049] | [0.050] |  |
| Ever had foreign postdoc | |  |  |  |  | 0.109* | 0.105* | 0.106* | 0.114** |  |
|  | |  |  |  |  | [0.043] | [0.043] | [0.043] | [0.044] |  |
| Additional Controls: (1) | |  |  |  |  |  |  |  |  |  |
| Age | | X | X | X | X | X | X | X | X | X |
| Foreign PhD | | X | X | X | X | X | X | X | X | X |
| Gender | | X | X | X | X | X | X | X | X | X |
| Application Year | | X | X | X | X | X | X | X | X | X |
| Undergraduate Variables | |  | X |  |  |  |  |  | X |  |
| Predoctoral Variables | |  |  | X |  |  |  |  | X | X |
| PhD Variables | |  |  |  | X |  |  |  | X |  |
| Postdoctoral Variables (1) | |  |  |  |  | X | X | X | X | X |
| Fellowship Variables | |  |  |  |  |  |  |  | X |  |
| Number of R01 Submissions | | |  |  |  |  |  |  | X |  |
| Observations | 2,397 | | 2,397 | 2,397 | 2,397 | 2,397 | 2,395 | 2,395 | 2,395 | 1,114 |

Robust standard errors in brackets. *** p<0.001, ** p<0.01, * p<0.05. 1) See Table S2 for full list of variables in these categories. 2) 2 Observations dropped because of collinearity. Source: NIH IMPAC II, National Science Foundation Doctoral Record File, American Association of Medical Colleges faculty roster, select NIH Biosketches, Web of Science®.

**Table D. Contribution of Covariates to Explained Portion of the Black NIH Funding Gap**

|  | **Main Estimate** | **Biosketch Model** | **Explained** | **Percentage of Base** |
| --- | --- | --- | --- | --- |
| **Black** | -0.141*** | -0.084*** | -0.057*** | -40.4% |
|  | [0.023] | [0.024] * | [0.011] |  |
| **Biosketch Publications** | No | Yes | -0.048*** | -34.0% |
|  |  |  | [.008] |  |
| **Biosketch Grants** | No | Yes | -0.007 | -5.0% |
|  |  |  | [0.005] |  |
| **Biosketch Scholarly** | No | Yes | 0.001 | 0.7% |
| **Activities** |  |  | [0.006] |  |
| **Biosketch Academic** | No | Yes | -0.004 | -2.8% |
| **Rank** |  |  | [0.003] |  |
|  |  |  |  |  |
|  | **Main Estimate** | **Full Model** | **Explained** | **Percentage**  **of Base** |
| **Black** | -0.141*** | -0.067** | -0.073*** | -51.8% |
|  | [0.023] | [0.022] | [0.011] |  |
| **Biosketch Publications** | No | Yes | -0.036*** | -25.5% |
|  |  |  | [.009] |  |
| **NIH Grants** | No | Yes | -0.028*** | -19.9% |
|  |  |  | [0.007] |  |
| **Employer** | No | Yes | -0.005 | -3.5% |
|  |  |  | [0.003] |  |
| **NIH ICs** | No | Yes | -0.004 | -2.8% |
|  |  |  | [0.005] |  |
|  |  |  |  |  |

Source: NIH IMPAC II, National Science Foundation Doctoral Record File, American Association of Medical Colleges faculty roster, select NIH Biosketches, Web of Science®. Random Sample from Ginther et al. (2011) N = 2,397. Biosketch Model includes covariates from Main, Rank, Grants, Scholarly Activities, Field Normalized Publications, Coauthor Publications listed in Table S1. Full Model includes covariates listed in Table S1. Statistical significance: *** p<0.001, ** p<0.01, * p<0.05.

**Table E. Counts of Applications and Awards by Race and Investigator Status**

|  |  |  |  |  |
| --- | --- | --- | --- | --- |
|  | **Asian** | **Black** | **Hispanic** | **White** |
|  |  |  |  |  |
| **Full Sample** | 600 | 598 | 600 | 599 |
| Scored | 334 | 240 | 341 | 361 |
| R01 Award | 145 | 83 | 148 | 161 |
| Percent Scored | 55.7% | 40.1%*** | 56.8% | 60.3% |
| Percent Awarded | 24.2% | 13.9%*** | 24.7% | 26.9% |
|  |  |  |  |  |
| **New Investigator** |  |  |  |  |
| Total | 285 | 374 | 338 | 235 |
| Scored | 154 | 123 | 163 | 116 |
| R01 Award | 61 | 40 | 67 | 50 |
| Percent Scored | 54.0% | 32.9%*** | 48.2% | 49.4% |
| Percent Awarded | 21.4% | 10.7%*** | 19.8% | 21.3% |
|  |  |  |  |  |
| **Experienced Investigator** | |  |  |  |
| Total | 315 | 224 | 262 | 364 |
| Scored | 180 | 117 | 178 | 245 |
| R01 Award | 84 | 43 | 81 | 111 |
| Percent Scored | 57.1%** | 52.2%*** | 67.9% | 67.3% |
| Percent Awarded | 26.7% | 19.2%** | 30.9% | 30.5% |

Source: NIH IMPAC II, National Science Foundation Doctoral Record File, American Association of Medical Colleges faculty roster, select NIH Biosketches, Web of Science®. Random Sample from Ginther et al. (2011) N = 2,397. Significantly different from white investigators: *** p<0.001, ** p<0.01, * p<0.05.

**Table F. Average of Productivity Measures by Race/Ethnicity for New Investigators, NIH R01 Awardees and Non-awardees**

|  | R01 Awardees | | | |  | R01 Applicants/ Not Awarded | | | |
| --- | --- | --- | --- | --- | --- | --- | --- | --- | --- |
|  | Asian | Black | Hispanic | White |  | Asian | Black | Hispanic | White |
| Total Publications | 18.56 | 17.90 | 19.58 | 20.32 |  | 20.94 | 16.38*** | 19.00 | 19.87 |
|  | [8.46] | [8.60] | [7.74] | [9.23] |  | [9.82] | [10.61] | [10.61] | [10.55] |
| Total Citations | 958.02 | 478.6** | 1262.78 | 987.60 |  | 919.99* | 341.12*** | 670.68 | 678.82 |
|  | [1199.11] | [398.81] | [1417.59] | [1130.82] |  | [1141.79] | [380.65] | [858.36] | [669.94] |
| Sum of Journal Impact | 94.30 | 43.52*** | 102.30 | 84.40 |  | 88.95*** | 39.52*** | 68.45 | 63.64 |
| Factors | [54.79] | [31.83] | [89.37] | [57.26] |  | [65.74] | [40.16] | [59.46] | [56.05] |
| Total Number of Coauthors | 35.21 | 29.90 | 41.24 | 38.18 |  | 45.56 | 26.72*** | 34.61 | 35.30 |
|  | [21.22] | [21.23] | [27.81] | [24.69] |  | [67.65] | [27.83] | [25.60] | [28.59] |
| Percentage of Papers | 3.25 | 5.04 | 1.47* | 3.16 |  | 4.16 | 7.47* | 5.50 | 5.19 |
| Uncited | [5.33] | [7.05] | [2.86] | [5.69] |  | [8.13] | [12.53] | [10.10] | [10.54] |
| Percentage of Papers in | 69.89 | 59.91** | 71.83 | 72.70 |  | 64.48 | 54.38*** | 60.99* | 65.75 |
| Top Quartile of Field | [18.84] | [25.90] | [22.31] | [16.50] |  | [22.10] | [27.89] | [24.36] | [22.07] |
| Percentage of Papers among | 5.15 | 4.35 | 7.39 | 5.78 |  | 5.34 | 2.57*** | 4.12 | 4.89 |
| Top 10% of citations in field | [3.87] | [4.45] | [5.83] | [4.09] |  | [4.40] | [2.89] | [4.25] | [4.31] |
| Percentage of Coauthors Papers | 57.69 | 59.99 | 59.66 | 63.18 |  | 53.12 | 47.27** | 49.54* | 54.89 |
| in Top Quartile of Field | [20.58] | [24.22] | [17.57] | [17.06] |  | [21.98] | [27.67] | [24.52] | [23.84] |
| Single Authored / Total Papers | 0.02* | 0.07 | 0.04 | 0.05 |  | 0.02*** | 0.09 | 0.05 | 0.07 |
|  | [.07] | [.15] | [.08] | [.09] |  | [.06] | [.19] | [.16] | [.14] |
| First Authored / Total Papers | 0.44 | 0.32 | 0.37 | 0.39 |  | 0.39 | 0.32 | 0.33 | 0.35 |
|  | [.20] | [.20] | [.25] | [.18] |  | [.23] | [.26] | [.24] | [.22] |
| Last Authored / Total Papers | 0.11* | 0.16 | 0.14 | 0.17 |  | 0.15 | 0.14 | 0.14* | 0.17 |
|  | [.14] | [.22] | [.19] | [.18] |  | [.18] | [.19] | [.19] | [.17] |
| Maximum Impact Factor | 16.57 | 8.00** | 17.01 | 13.45 |  | 15.06** | 8.37*** | 13.00 | 11.62 |
|  | [11.97] | [7.24] | [11.61] | [9.99] |  | [11.14] | [8.97] | [12.22] | [10.85] |
| Median Impact Factor | 5.10* | 2.47** | 4.61 | 4.00 |  | 3.86*** | 2.39* | 3.17 | 2.93 |
|  | [3.00] | [1.97] | [3.36] | [2.88] |  | [2.60] | [2.95] | [2.45] | [2.47] |
| Average Publications during | 4.59 | 3.53 | 3.88 | 5.06 |  | 4.63* | 2.65** | 4.26 | 3.69 |
| PhD | [3.85] | [3.63] | [3.39] | [5.35] |  | [5.03} | [4.13] | [5.03] | [4.39] |
| Average Publications during | 5.26 | 3.33* | 4.99 | 4.92 |  | 4.29* | 2.64* | 3.97 | 3.34 |
| Postdoc | [4.65] | [3.13] | [4.80] | [3.95] |  | [4.34] | [3.31] | [4.76] | [3.72] |
| Average Publications during | 7.02 | 7.83 | 8.57 | 8.02 |  | 9.70 | 7.66** | 8.23* | 10.05 |
| Principal Investigator | [8.50] | [7.68] | [9.40] | [7.95] |  | [9.16] | [8.56] | [9.14] | [9.78] |
| Average Citations during | 340.39 | 146.80 | 264.52 | 243.24 |  | 267.90 | 87.63*** | 205.43 | 191.09 |
| PhD | [774.95] | [243.04] | [377.87] | [315.93] |  | [566.49] | [182.31] | [426.69] | [327.14] |
| Average Citations during | 356.52 | 135.63 | 418.45 | 363.38 |  | 270.24* | 92.22*** | 194.68 | 185.33 |
| Postdoc | [524.69] | [196.85] | [745.18] | [996.70] |  | [444.18] | [195.62] | [356.72] | [356.79] |
| Average Citations during | 257.11 | 195.80 | 579.42 | 380.58 |  | 372.85 | 160.96*** | 269.60 | 300.08 |
| Principal Investigator | [342.99] | [263.22] | [1037.53] | [604.66] |  | [810.96] | [246.53] | [567.84] | [383.92] |
| Average Prior R21 Awards | 0.13 | 0.15 | 0.10 | 0.06 |  | 0.11 | 0.07 | 0.07 | 0.06 |
|  | [.34] | [.36] | [.35] | [.24] |  | [.34] | [.29] | [.26] | [.26] |
| Average Prior R29 Awards | 0.07 | 0** | 0.10 | 0.12 |  | 0.06 | 0.03* | 0.04 | 0.07 |
|  | [.25] | [0] | [.31] | [.33] |  | [.24] | [.17] | [.19] | [.26] |
| Average Prior Other NIH Awards | 0.18 | 0.28 | 0.12 | 0.28 |  | 0.19 | 0.26 | 0.26 | 0.29 |
|  | [.50] | [.60] | [.37] | [.57] |  | [.58] | [.69] | [.87] | [.96] |
| Bachelor's Top 100 NIH Funding | 0.08** | 0.23 | 0.25 | 0.30 |  | 0.09*** | 0.22 | 0.18 | 0.22 |
|  | [.28] | [.42] | [.44] | [.46] |  | [.29] | [.42] | [.39] | [.41] |
| PhD Top 100 NIH Funding | 0.56 | 0.58 | 0.30*** | 0.60 |  | 0.45 | 0.48 | 0.40 | 0.43 |
|  | [.50] | [.50] | [.46] | [.49] |  | [.50] | [.50] | [.49] | [.50] |
| Postdoc Top 100 NIH Funding | 0.52 | 0.48 | 0.36 | 0.42 |  | 0.44 | 0.38 | 0.44 | 0.41 |
|  | [.50] | [.51] | [.48] | [.50] |  | [.50] | [.49] | [.50] | [.49] |
| Employer Top 100 NIH Funding | 0.70 | 0.73 | 0.70 | 0.74 |  | 0.64 | 0.50* | 0.64 | 0.59 |
|  | [.46] | [.45] | [.46] | [.44] |  | [.48] | [.50] | [.48] | [.49] |
| Observations | 61 | 40 | 67 | 50 |  | 224 | 334 | 271 | 185 |

*** p< .001, ** p < .01, * p<.05 compared with whites.

**Table G. Average of Productivity Measures by Race/Ethnicity for Experienced Investigators, NIH R01 Awardees and Non-awardees**

|  | R01 Awardees | | | |  | R01 Applicants/ Not Awarded | | | |
| --- | --- | --- | --- | --- | --- | --- | --- | --- | --- |
|  | Asian | Black | Hispanic | White |  | Asian | Black | Hispanic | White |
| Total Publications | 26.85 | 25.51 | 27.94 | 27.50 |  | 28.71* | 21.70*** | 26.28 | 26.36 |
|  | [11.26] | [12.20] | [11.61] | [10.97] |  | [11.94] | [10.18] | [9.94] | [12.08] |
| Total Citations | 1399.14 | 1063.33 | 1499.68 | 1613.02 |  | 1724.06 | 687.92*** | 1290.24 | 1425.21 |
|  | [1035.41] | [1229.49] | [1171.88] | [1696.68] |  | [1955.09] | [662.47] | [1320.18] | [1970.81] |
| Sum of Journal Impact | 154.22 | 84.96** | 136.45 | 141.54 |  | 152.97*** | 57.63*** | 118.87 | 110.93 |
| Factors | [113.92] | [106.37] | [89.44] | [108.06] |  | [116.84] | [45.40] | [93.70] | [86.01] |
| Total Number of Coauthors | 53.06 | 44.86 | 52.90 | 52.16 |  | 57.91** | 36.90*** | 51.82 | 49.57 |
|  | [28.92] | [43.63] | [27.41] | [39.68] |  | [34.73] | [23.79] | [27.00] | [37.64] |
| Percentage of Papers | 2.98 | 4.21 | 2.38 | 3.13 |  | 4.25 | 4.68 | 2.97 | 3.33 |
| Uncited | [6.32] | [5.66] | [3.90] | [5.27] |  | [6.29] | [9.90] | [4.94] | [7.08] |
| Percentage of Papers in | 70.56 | 67.89 | 73.13 | 70.26 |  | 67.74 | 58.21*** | 69.53 | 66.87 |
| Top Quartile of Field | [21.26] | [20.14] | [19.85] | [23.77] |  | [19.87] | [25.41] | [20.08] | [24.03] |
| Percentage of Papers among | 8.67 | 6.51* | 9.41 | 9.23 |  | 9.14 | 4.35*** | 8.15 | 8.28 |
| Top 10% of citations in field | [5.68] | [5.78] | [5.75] | [7.07] |  | [7.09] | [3.64] | [5.76] | [5.83] |
| Percentage of Coauthors Papers | 57.67 | 57.39 | 58.59 | 57.29 |  | 54.40 | 47.83 | 52.63 | 51.92 |
| in Top Quartile of Field | [20.99] | [19.74] | [20.21] | [22.05] |  | [21.63] | [24.86] | [19.97] | [22.33] |
| Single Authored / Total Papers | 0.02 | 0.07** | 0.03 | 0.03 |  | 0.03** | 0.07* | 0.03** | 0.05 |
|  | [.04] | [.11] | [.06] | [.05] |  | [.06] | [.14] | [.06] | [.08] |
| First Authored / Total Papers | 0.30*** | 0.35*** | 0.23 | 0.20 |  | 0.26 | 0.26 | 0.25 | 0.23 |
|  | [.24] | [.22] | [.18] | [.18] |  | [.21] | [.22] | [.21] | [.20] |
| Last Authored / Total Papers | 0.31 | 0.23** | 0.32 | 0.38 |  | 0.34 | 0.27*** | 0.32 | 0.35 |
|  | [.26] | [.26] | [.23] | [.27] |  | [.26] | [.24] | [.26] | [.26] |
| Maximum Impact Factor | 19.83 | 12.29* | 17.63 | 17.72 |  | 19.49*** | 8.83*** | 14.89 | 14.83 |
|  | [13.84] | [9.76] | [11.98] | [12.68] |  | [12.99] | [7.67] | [10.72] | [11.01] |
| Median Impact Factor | 5.46* | 2.57*** | 4.26 | 4.44 |  | 4.68*** | 2.24*** | 4.16 | 3.64 |
|  | [3.52] | [1.97] | [2.68] | [3.56] |  | [3.01] | [1.70] | [2.96] | [2.76] |
| Average Publications during | 2.74*** | 1.72 | 2.88*** | 1.21 |  | 2.87*** | 1.58 | 2.25 | 1.77 |
| PhD | [3.93] | [3.10] | [3.80] | [2.34] |  | [3.99] | [2.84] | [3.67] | [3.41] |
| Average Publications during | 3.30* | 1.56 | 2.77 | 2.00 |  | 3.17*** | 1.66 | 2.82* | 1.94 |
| Postdoc | [4.03] | [2.42] | [3.49] | [3.31] |  | [4.13] | [2.46] | [4.55] | [3.30] |
| Average Publications during | 18.18 | 18.40 | 19.75 | 21.39 |  | 19.98 | 15.13*** | 18.85 | 19.83 |
| Principal Investigator | [12.82] | [12.42] | [11.68] | [10.88] |  | [12.40] | [9.73] | [11.23] | [11.09] |
| Average Citations during | 221.05* | 56.77 | 225.78* | 103.47 |  | 252.79 | 75.93 | 172.44 | 252.10 |
| PhD | [436.59] | [129.73] | [473.79] | [250.09] |  | [772.77] | [202.05] | [487.39] | [1459.07] |
| Average Citations during | 286.05 | 71.60* | 271.43 | 198.41 |  | 365.00 | 84.93* | 218.36 | 230.13 |
| Postdoc | [469.28] | [145.63] | [450.61] | [365.08] |  | [858.43] | [158.00] | [411.48] | [876.17] |
| Average Citations during | 895.94* | 934.95 | 1009.89 | 1309.78 |  | 1108.96 | 527.06*** | 890.51 | 942.39 |
| Principal Investigator | [893.45] | [1223.88] | [903.49] | [1548.68] |  | [1307.15] | [604.05] | [1060.33] | [863.74] |
| Average Prior R01 Awards | 3.10** | 3.07* | 2.67*** | 4.45 |  | 3.05** | 2.27*** | 3.07** | 3.96 |
|  | [3.22] | [3.23] | [2.24] | [3.46] |  | [3.21] | [2.29] | [2.83] | [3.98] |
| Average Prior R21 Awards | 0.17 | 0.16 | 0.21 | 0.18 |  | 0.18 | 0.23 | 0.25 | 0.17 |
|  | [.41] | [.48] | [.56] | [.54] |  | [.44] | [.49] | [.54] | [.43] |
| Average Prior R29 Awards | 0.20 | 0.09 | 0.17 | 0.21 |  | 0.16 | 0.09 | 0.09 | 0.11 |
|  | [.40] | [.29] | [.38] | [.41] |  | [.36] | [.29] | [.28] | [.32] |
| Average Prior Other NIH Awards | 0.40 | 0.47 | 0.36 | 0.53 |  | 0.45 | 0.44 | 0.35 | 0.41 |
|  | [.78] | [.85] | [.58] | [.91] |  | [.95] | [.97] | [.62] | [.77] |
| Average Prior Type 2 R01 Awards | 1.94* | 1.37** | 1.74** | 2.84 |  | 1.45 | 0.74*** | 1.56 | 1.71 |
|  | [2.51] | [1.88] | [1.80] | [3.18] |  | [2.15] | [1.31] | [2.06] | [2.38] |
| Bachelor's Top 100 NIH Funding | 0.11*** | 0.33 | 0.22 | 0.34 |  | 0.11*** | 0.22*** | 0.23*** | 0.38 |
|  | [.31] | [.47] | [.42] | [.48] |  | [.32] | [.42] | [.42] | [.49] |
| PhD Top 100 NIH Funding | 0.49 | 0.60 | 0.35** | 0.53 |  | 0.46 | 0.48 | 0.42* | 0.53 |
|  | [.50] | [.49] | [.48] | [.50] |  | [.50] | [.50] | [.49] | [.50] |
|  |  |  |  |  |  |  |  |  |  |
|  |  | R01 Awardees | |  |  |  | Not Awarded | |  |
| Table S7, Continued: | Asian | Black | Hispanic | White |  | Asian | Black | Hispanic | White |
| Postdoc Top 100 NIH Funding | 0.49 | 0.26 | 0.41 | 0.48 |  | 0.44 | 0.41 | 0.44 | 0.49 |
|  | [.50] | [.44] | [.49] | [.50] |  | [.50] | [.49] | [.50] | [.50] |
| Employer Top 100 NIH Funding | 0.81 | 0.79 | 0.73 | 0.79 |  | 0.74 | 0.73 | 0.80 | 0.75 |
|  | [.40] | [.41] | [.45] | [.41] |  | [.44] | [.45] | [.40] | [.43] |
| Observations | 84 | 43 | 81 | 111 |  | 231 | 181 | 181 | 253 |

*** p< .001, ** p < .01, * p<.05 compared with whites.

**References and notes**

1. Ginther DK, Schaffer WT, Schnell J, Masimore B, Liu F, Haak LL, Kington R.. Race, ethnicity, and NIH research awards. Science. 2011;333:1015-19.
2. A complete description of the data for Ginther et al. [1] is available online at: <http://www.sciencemag.org/content/333/6045/1015/suppl/DC1>.
3. Wooldridge JM. Econometric Analysis of Cross Section and Panel Data. 2nd ed. Cambridge (MA): MIT Press; 2010.
4. The Carnegie Classification of Institutions of Higher Education, <http://carnegieclassifications.iu.edu/>.
5. US Department of Education, White House Initiative on Historically Black Colleges and Universities School Directory, <https://sites.ed.gov/whhbcu/>.
6. The Oberlin Group of Libraries, Oberlin Group Institution Members, <http://www.oberlingroup.org/group-members>.
7. 3,240 publications do not have impact factors because either the journal was not indexed in Web of Science or the paper was published before 1994 (before the time impact factors are available electronically).
8. For more information see, Using Bibliometrics in Evaluating Research, <http://wokinfo.com/media/mtrp/UsingBibliometricsinEval_WP.pdf>.
9. Clarivate Analytics, Journal Citation Reports, http://clarivate.com/?product=journal-citation-reports.

1. AFFILIATIONS:

   * Author for correspondence. E-mail: dginther@ku.edu.

   Department of Economics and Center for Science, Technology & Economic Policy, Institute for Policy & Social Research, University of Kansas, Lawrence, KS 66045, USA and National Bureau of Economic Research, Cambridge, MA 02138 USA. [↑](#footnote-ref-1)
2. Discovery Logic/Clarivate Analytics, Rockville, MD 20850, USA. [↑](#footnote-ref-2)
3. Grinnell College, Grinnell, IA 50112, USA. [↑](#footnote-ref-3)
4. National Institutes of Health, Bethesda, MD 20892, USA. [↑](#footnote-ref-4)
